# Alleviating Recombinant Tissue Plasminogen Activator‐induced Hemorrhagic Transformation in Ischemic Stroke via Targeted Delivery of a Ferroptosis Inhibitor

**DOI:** 10.1002/advs.202309517

**Published:** 2024-04-22

**Authors:** Yan‐Qin Geng, Li‐Na Qiu, Yuan‐Qiu Cheng, Juan‐Juan Li, Yi‐Lin Ma, Cheng‐Cheng Zhao, Ying Cai, Xue‐Bin Zhang, Jieli Chen, Yu‐Chen Pan, Ke‐Rang Wang, Xiu‐Hua Yao, Dong‐Sheng Guo, Jia‐Ling Wu

**Affiliations:** ^1^ School of Medicine Nankai University Tianjin 300071 China; ^2^ Tianjin Huanhu Hospital Tianjin 300350 China; ^3^ Department of Neurology Tianjin Huanhu Hospital Tianjin 300350 China; ^4^ Tianjin Key Laboratory of Cerebral Vascular and Neurodegenerative Diseases Tianjin Neurosurgical Institute Tianjin Huanhu Hospital Tianjin 300350 China; ^5^ College of Chemistry State Key Laboratory of Elemento‐Organic Chemistry Key Laboratory of Functional Polymer Materials (Ministry of Education) Frontiers Science Center for New Organic Matter Collaborative Innovation Center of Chemical Science and Engineering (Tianjin) Nankai University Tianjin 300071 China; ^6^ College of Chemistry and Environmental Science Key Laboratory of Medicinal Chemistry and Molecular Diagnosis (Ministry of Education) Key Laboratory of Chemical Biology of Hebei Province Hebei University Baoding 071002 China; ^7^ Clinical College of Neurology Neurosurgery and Neurorehabilitation Tianjin Medical University Tianjin 300071 China; ^8^ Department of Pathology Tianjin Huanhu Hospital Tianjin 300350 China; ^9^ Xinjiang Key Laboratory of Novel Functional Materials Chemistry College of Chemistry and Environmental Sciences Kashi University Kashi 844000 China

**Keywords:** drug delivery, ferroptosis, ischemic stroke, macrocyclic receptor, supramolecular materials

## Abstract

Intravenous thrombolysis with recombinant tissue plasminogen activator (rtPA) is the primary treatment for ischemic stroke. However, rtPA treatment can substantially increase blood‐brain barrier (BBB) permeability and susceptibility to hemorrhagic transformation. Herein, the mechanism underlying the side effects of rtPA treatment is investigated and demonstrated that ferroptosis plays an important role. The ferroptosis inhibitor, liproxstatin‐1 (Lip) is proposed to alleviate the side effects. A well‐designed macrocyclic carrier, glucose‐modified azocalix[4]arene (GluAC4A), is prepared to deliver Lip to the ischemic site. GluAC4A bound tightly to Lip and markedly improved its solubility. Glucose, modified at the upper rim of GluAC4A, imparts BBB targeting to the drug delivery system owing to the presence of glucose transporter 1 on the BBB surface. The responsiveness of GluAC4A to hypoxia due to the presence of azo groups enabled the targeted release of Lip at the ischemic site. GluAC4A successfully improved drug accumulation in the brain, and Lip@GluAC4A significantly reduced ferroptosis, BBB leakage, and neurological deficits induced by rtPA in vivo. These findings deepen the understanding of the side effects of rtPA treatment and provide a novel strategy for their effective mitigation, which is of great significance for the treatment and prognosis of patients with ischemic stroke.

## Graphical abstract

Alleviating hemorrhagic transformation due to blood‐brain barrier disruption following recombinant tissue plasminogen activator (rtPA) therapy remains a significant concern in ischemic stroke treatment. Herein, a novel mechanism underlying rtPA‐induced side effects are identified, that is, the induction of ferroptosis in astrocytes, and proposed a new treatment paradigm using a ferroptosis inhibitor liprostatin‐1 (Lip). To address challenges in delivering Lip to the brain, a targeted macrocyclic carrier, glucose‐modified azocalix[4]arene (GluAC4A), is developed, and the effectiveness of Lip@GluAC4A in treating rtPA‐induced side effects is verified.

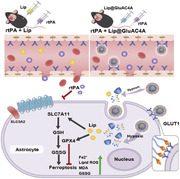

## 1 Introduction

Acute ischemic stroke (AIS) is one of the foremost causes of disability and mortality worldwide.^[^ ^1^ ^]^ Intravenous recombinant tissue plasminogen activator (rtPA) thrombolysis has emerged as a critical intervention for ischemic stroke, significantly improving patient outcomes by enabling early recanalization of occluded blood vessels and reducing risks of disability and mortality.^[^ ^2^ ^]^ However, rtPA treatment substantially increases blood‐brain barrier (BBB) permeability and susceptibility to hemorrhagic transformation, presenting significant challenges.^[^ ^3^ ^]^ Notably, the extended use of rtPA beyond the conventional period amplifies the risk of hemorrhagic transformation;^[^ ^3^, ^4^ ^]^ additionally, cerebral hemorrhage can further intensify ferroptosis, a form of regulated cell death driven by lethal iron‐catalyzed lipid damage.^[^ ^5^ ^]^ Iron homeostasis is perturbed in the brain following ischemic stroke, resulting in intracellular iron overload, which is a major inducer of ferroptosis after cerebral ischemia. The inhibition of iron overload mitigates ferroptosis and reduces injury in ischemic stroke.^[^ ^6^ ^]^ Introducing ferroptosis inhibitors into rtPA therapy may be an effective method to alleviate the side effects of rtPA and improve outcomes in patients with ischemic stroke. However, related research is scarce, and in‐depth studies are urgently required.

Liproxstatin‐1 (Lip), a recently identified ferroptosis inhibitor, showed promise in preventing lipid reactive oxygen species (ROS) accumulation and mitigating erastin‐induced ferroptosis.^[^ ^7^ ^]^ Thus, it has been used for the treatment of hepatic ischemia‐reperfusion injury,^[^ ^8^ ^]^ spontaneous pancreatic ductal adenocarcinoma,^[^ ^9^ ^]^ and traumatic brain injury.^[^ ^10^ ^]^ Presently, the methods for Lip administration are predominantly intranasal^[^ ^11^ ^]^ and intraperitoneal,^[^ ^12^ ^]^ which are limited by low bioavailability and slow onset.^[^ ^13^ ^]^ Intravenous administration, which is commonly used to ensure 100% bioavailability with rapid onset, is considered optimal.^[^ ^14^ ^]^ However, the intravenous administration of Lip is challenging owing to its low solubility and inability to penetrate the BBB. Therefore, it is imperative to develop a carrier that can enhance the solubility of Lip, facilitate its transport across the BBB, and achieve its targeted release at the lesion site. Moreover, from a treatment perspective, drug delivery systems should be simple and reproducible.^[^ ^15^ ^]^

Macrocyclic receptors, such as cyclodextrins, calixarenes, and cucurbiturils, have been extensively employed in biomedicine for various purposes, including drug delivery.^[^ ^15^, ^16^ ^]^ Macrocyclic carriers load drugs via host‐guest recognition, and therefore, have the following advantages. i) Benefiting from the dynamic nature of host‐guest recognition, drugs can be loaded into the cavity of carriers by simple mixing, minimizing the uncontrollable factors in the preparation process.^[^ ^16^, ^17^ ^]^ ii) Host‐guest recognition provides defined stoichiometry and characteristic binding affinity, enabling quantitative loading and ratiometric delivery of drugs under thermodynamic control and ensuring good batch‐to‐batch reproducibility.^[^ ^18^ ^]^ iii) Macrocyclic carriers provide protection to drugs at a molecular level and therefore, can effectively regulate their physicochemical properties, such as solubility.^[^ ^18^, ^19^ ^]^ Consequently, macrocyclic carriers are promising candidates for simple and reproducible drug delivery systems. Among various macrocycles, calixarenes are considered to have almost unlimited possibilities regarding structure and application because they are highly modifiable.^[^ ^20^ ^]^ Through modifications, calixarenes can tightly bind guests and respond to stimuli and/or targets.^[^ ^17^, ^19^, ^21^ ^]^ Therefore, we envisage that functionalized calixarenes may pave the way for delivering ferroptosis inhibitors that can alleviate rtPA‐induced hemorrhagic transformation in ischemic stroke.

**Scheme 1:**
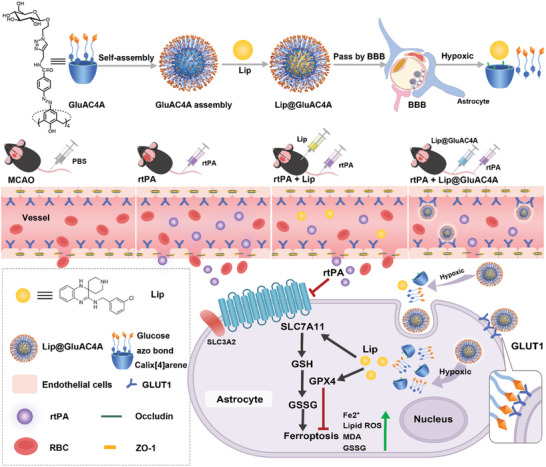
Schematic illustration of Lip@GluAC4A and its therapeutic mechanism in rtPA‐induced hemorrhagic transformation.

Herein, we initially investigated whether rtPA treatment could induce ferroptosis‐related changes in a mouse model of middle cerebral artery occlusion (MCAO), which served as the foundation for further research. Subsequently, we explored the mitigating effects of Lip on rtPA‐induced side effects in an in vitro model. To effectively deliver Lip and release it at the lesion site via intravenous administration, we designed and synthesized a macrocyclic carrier called glucose‐modified azocalix[4]arene (GluAC4A, **Scheme** **1**). GluAC4A exhibited a strong binding affinity for Lip and significantly improved its solubility. The presence of a glucose modification on the upper rim of GluAC4A facilitated BBB targeting owing to the expression of glucose transporter 1 (GLUT1) on the BBB surface. Additionally, GluAC4A demonstrated responsiveness to hypoxia through azo groups, enabling the targeted release of Lip specifically in ischemic brain tissue after MCAO. We conducted a systematic in vivo study to investigate the mitigating effects of Lip@GluAC4A on the side effects of rtPA regarding neurological function, BBB integrity, hemorrhagic transformation, and ferroptosis. Furthermore, we conducted mechanistic studies to elucidate the function of Lip@GluAC4A.

## 2 Results and Discussion

### 2.1 rtPA‐Induced Ferroptosis in Astrocytes in a MCAO Mouse Model

**Figure 1:**
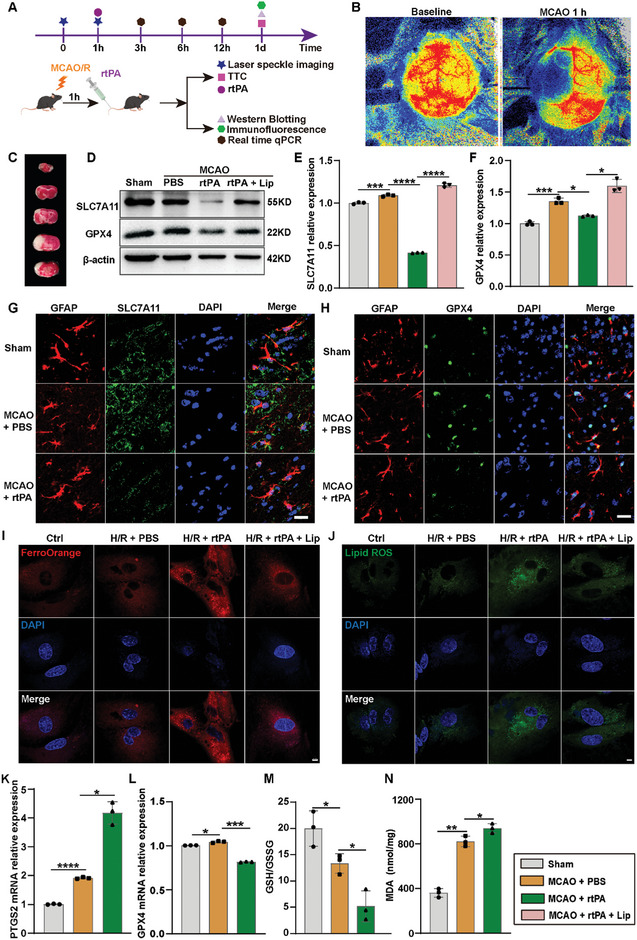
rtPA‐induced ferroptosis in astrocytes in a MCAO mouse model. A) Experimental timeline. B) Changes in the basal cerebral blood flow and blood flow after cerebral infarction in C57BL/6J male mice. C) Brain tissue TTC staining after stroke. D–F) Western blot images depicting the expression of SLC7A11 and GPX4 in MCAO mice of different groups (Sham, MCAO + PBS, MCAO + rtPA; *n* = 3/group). G,H) Immunofluorescence was used to assess the expression of SLC7A11 and GPX4 in astrocytes collected from sham‐treated mice and mice treated with or without 10 mg kg^−1^ rtPA 24 h after MCAO; Scale bars: 20 µm. I,J) Representative confocal images showing Fe^2+^ (I, *n* = 4/group) and lipid ROS (J, *n* = 4/group) in astrocytes treated with H/R + PBS, H/R + rtPA, and H/R + rtPA + Lip. Scale bars: 5 µm. K‐N. PTGS2 mRNA levels (K), GPX4 mRNA levels (L), the GSH/GSSG ratio (M), and MDA levels (N) in the ischemic brains of mice treated with PBS and rtPA 24 h after MCAO. *n* = 3/group. Data represent the mean ± SD, ^*^ *p* < 0.05, ^**^ *p* < 0.01, ^***^ *p* < 0.001, ^****^ *p* < 0.0001.

We conducted a series of experiments to ascertain the occurrence of ferroptosis in mice following MCAO and understand its relationship with rtPA treatment (**Figure** **1**A). First, we successfully prepared the MCAO mouse model. The laser speckle flow map indicated that cerebral blood flow decreased significantly 1 h after MCAO, and triphenyl tetrazolium chloride (TTC) staining confirmed the presence of cerebral ischemic foci in the mice (Figure 1B,C). Then, we evaluated the expression levels of ferroptosis‐related proteins and genes under ischemic conditions. Two pivotal antiferroptotic factors, solute carrier family seven member 11 (SLC7A11) and glutathione peroxidase 4 (GPX4),^[^ ^22^ ^]^ were examined. The expression levels of SLC7A11 and GPX4 were higher in ischemic brains than in normal brains (Figure 1D–F). Notably, following rtPA treatment, the levels of SLC7A11 and GPX4 decreased compared with those in the MCAO untreated group. Furthermore, the expression levels of SLC7A11 and GPX4 were restored after Lip administration, suggesting that rtPA might trigger ferroptosis by modulating the expression of key proteins involved in the ferroptosis pathway.

Subsequently, we conducted an immunofluorescence analysis to identify the types of brain cells expressing SLC7A11 and GPX4. Interestingly, SLC7A11 was widely expressed throughout the brain tissue, whereas GPX4 exhibited high co‐localization with GFAP^+^ astrocytes and limited co‐localization with NeuN^+^ neurons in the cerebral cortex of the mouse brain (Figure 1G,H; Figure S1, Supporting Information). To further demonstrate that rtPA can induce ferroptosis in MCAO mice, primarily in astrocytes, we isolated primary astrocytes from MCAO mice treated with rtPA after hypoxia/reoxygenation (H/R) and measured the levels of Fe^2+^ and lipid ROS. FerroOrange, a fluorescent probe, was used to detect unstable Fe^2+^ ions ^[^ ^23^ ^]^ whereas BODIPY 581/591 C11, a lipophilic dye, was used to measure lipid ROS levels.^[^ ^24^ ^]^ We observed that the levels of Fe^2+^ and lipid peroxides increased in astrocytes after rtPA treatment, and Lip alleviated the levels of Fe^2+^ and lipid ROS (Figure 1I,J). These findings suggest that astrocytes are involved in ferroptosis in MCAO mice treated with rtPA.

Additionally, we quantified the levels of prostaglandin‐endoperoxide synthase (PTGS2), GPX4, glutathione (GSH), and malondialdehyde (MDA) in ischemic brain tissue following MCAO. PTGS2 mRNA is a well‐known ferroptosis marker.^[^ ^25^ ^]^ To better demonstrate the occurrence and peak time of ferroptosis in vivo, we evaluated the changes in PTGS2 mRNA levels in MCAO mice at 3, 6, 12, and 24 h after rtPA treatment. The results revealed that the most significant alterations in PTGS2 mRNA levels occurred at 3 h after rtPA treatment (Figure S2, Supporting Information). PTGS2 mRNA expression increased following MCAO, and a further increase was observed after rtPA treatment (Figure 1K). This finding aligns with those of previous studies on ferroptosis‐related changes.^[^ ^12^, ^24^, ^26^ ^]^ Moreover, immunofluorescence staining of brain tissue showed that cyclooxygenase 2 (COX2) co‐localized with GFAP or S100 (Figure S3, Supporting Information). We employed S100β, in addition to GFAP, to allow a more comprehensive characterization of astrocytes. S100β is known to label the cell bodies of smaller astrocytes with less extended branching, complementing the information obtained from GFAP staining.^[^ ^27^ ^]^ This approach allowed us to obtain a more nuanced understanding of astrocyte morphology and function. Extracellular cystine can enter cells via SLC7A11 and is converted to cysteine through reduction for GSH synthesis.^[^ ^28^ ^]^ GPX4 utilizes GSH to reduce lipid hydroperoxides to lipid alcohols, inhibiting ferroptosis.^[^ ^29^ ^]^ Lipid peroxidation plays a central role in driving ferroptosis, and disruption of BBB integrity after ischemic stroke enables excess Fe^2+^ to generate ROS and promote lipid peroxidation, inducing ferroptosis.^[^ ^30^ ^]^ Therefore, we examined the effect of rtPA on GPX4 mRNA, GSH levels, and MDA (a lipid oxidation indicator) levels. We observed that rtPA treatment reduced GPX4 mRNA levels and diminished the GSH/GSSG ratio while concurrently increasing MDA levels (Figure 1L–N). These findings suggest that rtPA treatment contributes to the development of ferroptosis by affecting the SLC7A11‐GSH‐GPX4 pathway, potentially rendering the brain more susceptible to ferroptosis after MCAO.

### 2.2 Lip Alleviates rtPA‐Induced BBB Disruption by Inhibiting Ferroptosis In Vitro

**Figure 2:**
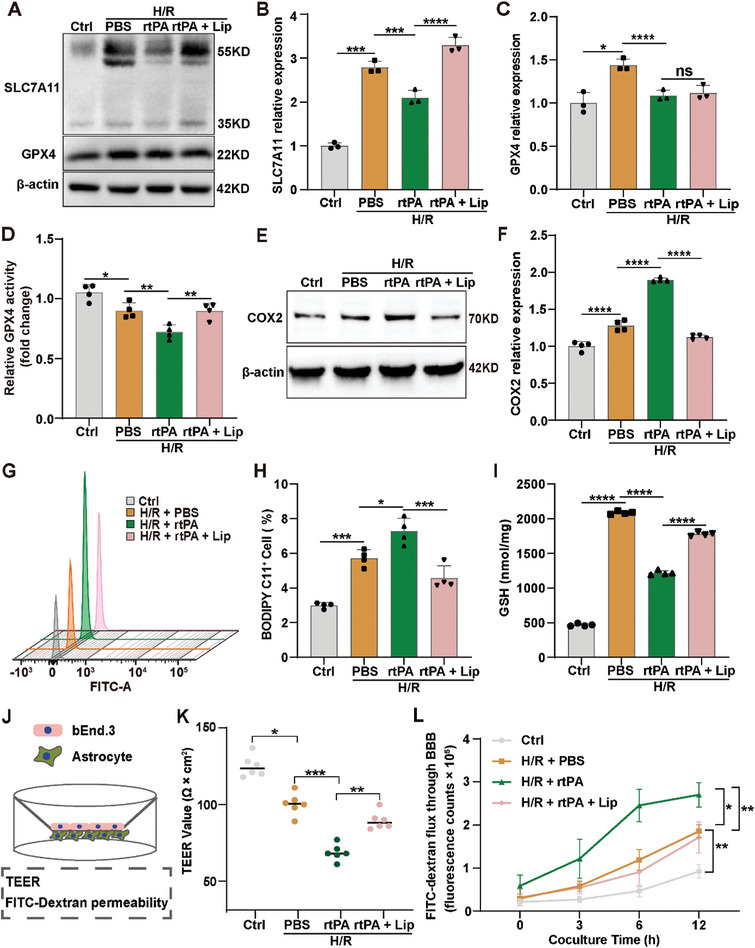
Lip alleviates rtPA‐induced BBB disruption by inhibiting ferroptosis in vitro. A–C) Western blot images depicting SLC7A11 and GPX4 expression in astrocytes of different groups (*n* = 3/group). D) GPX4 activity in astrocytes treated with H/R + PBS, H/R + rtPA, and H/R + rtPA + Lip (*n* = 4/group). E,F) Western blot images depicting COX2 expression in astrocytes of different groups (*n* = 4/group). G,H) Cell lipid peroxidation was assessed by BODIPY 581/591 C11 staining using flow cytometry (*n* = 4/group). I) GSH (I) levels in astrocytes treated with H/R + PBS, H/R + rtPA, and H/R + rtPA + Lip (*n* = 4/group). J–L) Schematic representation of the in vitro BBB model (J). TEER (K, *n* = 6/group) and FITC‐dextran permeability (L, *n* = 6/group) in astrocytes treated with H/R + PBS, H/R + rtPA, and H/R + rtPA + Lip. Data are presented as the mean ± SD, ^*^ *p* < 0.05, ^**^ *p* < 0.01, ^***^ *p* < 0.001, ^****^ *p* < 0.0001.

We conducted experiments in an in vitro model to demonstrate the effect of Lip in the attenuation of rtPA‐induced side effects. We observed an increase in SLC7A11 and GPX4 expression in astrocytes subjected to H/R, which subsequently decreased after rtPA treatment (**Figure** **2**A–C). This finding is consistent with those from the in vivo model, suggesting that SLC7A11 and GPX4 are involved in ferroptosis as key regulators in the in vitro model. We then administered Lip, a potent and specific ferroptosis inhibitor previously shown to attenuate H/R damage, to investigate whether the inhibition of ferroptosis could ameliorate brain injury.^[^ ^7^, ^11^ ^]^

Interestingly, while Lip treatment led to an increase in SLC7A11 expression, the protein level of GPX4 remained relatively unchanged after Lip application (Figure 2A–C). This led us to investigate whether Lip influenced GPX4 activity. Our analysis revealed that Lip treatment increased GPX4 activity in rtPA‐treated astrocytes (Figure 2D). In addition, we examined the protein expression from PTGS2 mRNA, a marker of ferroptosis, and observed that rtPA treatment increased the expression of COX2 (the PTGS2 protein) in astrocytes after hypoxia, and its levels decreased after Lip application (Figure 2E,F). Furthermore, Lip reduced the intensity of green fluorescence of BODIPY 581/591 C11 after H/R and rtPA treatments, suggesting its ability to prevent lipid peroxidation (Figure 2G,H). Unlike in the in vivo model, GSH levels increased in the in vitro H/R treatment model, possibly as a compensatory response. However, following rtPA treatment, GSH levels decreased, which was reversed by the application of Lip (Figure 2I). These results suggest that Lip attenuates ferroptosis‐related changes induced by rtPA.

Additionally, we established an in vitro BBB model and evaluated the effects of ischemia and rtPA on BBB permeability by measuring transepithelial electrical resistance (TEER) and fluorescein isothiocyanate‐dextran (FITC)‐dextran permeability. We observed that rtPA disrupted the BBB, whereas the ferroptosis inhibitor Lip mitigated this effect (Figure 2J–L). In summary, our findings confirm the involvement of ferroptosis in MCAO mice treated with rtPA and demonstrate that Lip mitigates the side effects of rtPA and restores BBB function by inhibiting ferroptosis.

### 2.3 The Design and Construction of the Supramolecular Drug Delivery System Lip@GluAC4A

Although Lip has the potential to alleviate rtPA‐induced BBB disruption, delivering Lip to the brain remains challenging. Therefore, there is an urgent need to develop novel drug carriers. Calixarene was selected as the macrocyclic scaffold because of its ease of modification and biocompatibility.^[^ ^16^, ^31^ ^]^ Additionally, the introduction of azo groups, which can be reduced by overexpressed azoreductase in hypoxic microenvironments, can confer hypoxia responsiveness.^[^ ^17^, ^32^ ^]^ Moreover, the deepened cavity of azocalixarene significantly enhances its drug‐binding affinity, thereby avoiding unwarranted off‐target leakage in blood circulation.^[^ ^17^, ^33^ ^]^ A glucose moiety can be attached to the upper rim of the azocalixarene, enabling BBB targeting due to the presence of GLUT1 on the BBB surface.^[^ ^34^ ^]^ Simultaneously, the introduction of glucose deepens the cavity of the calixarenes, provides new anchoring points, and may enhance the solubility and biocompatibility of azocalixarenes. Collectively, these principles guided the synthesis of GluAC4A.

**Figure 3:**
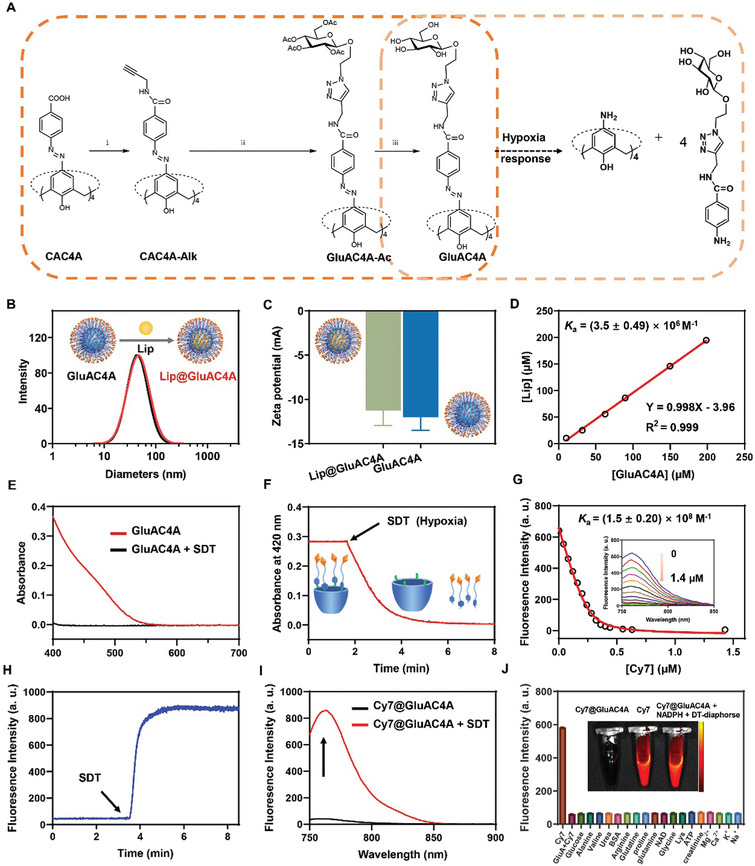
The design and construction of the supramolecular drug delivery system Lip@GluAC4A. A) The synthetic route of GluAC4A. (i) Propargylamine, HATU, DIPEA, DMF, rt; (ii) Glu‐N_3_, THF/H_2_O, CuSO_4_⋅5H_2_O, L‐ascorbic acid sodium salt, 55 °C; (iii) CH_3_OH, CH_3_ONa, rt. B) DLS results of Lip@GluAC4A and GluAC4A (1.0 mm). C) Zeta potential measurement of Lip@GluAC4A (0.10 mm) and GluAC4A (0.10 mm). D) Phase solubility diagram illustrating the interaction between Lip and GluAC4A in PBS (10 mM, pH 7.4) at 25 °C. E) Absorbance spectra of GluAC4A (10 µm) before and after the reduction by SDT (10 mm) in PBS (10 mm, pH 7.4) at 37 °C. F) Absorbance of GluAC4A (10 µm) at 420 nm as a function of time following the addition of SDT (10 mm) in PBS (10 mm, pH 7.4) at 37 °C. G) Direct fluorescence titration of Cy7 (0.50 µm, *λ*_ex_  =  736 nm, *λ*_em_  =  760 nm) with GluAC4A (up to 1.4 µm) in PBS (10 mm, pH 7.4) at 25 °C and the corresponding titration curve fitting, demonstrating a 1:1 binding stoichiometry. H) Fluorescence intensity of Cy7@GluAC4A (10/12 µm) at 760 nm as a function of time following the addition of SDT (10 mm) in PBS (10 mm, pH 7.4) at 37 °C. I) The release of Cy7@GluAC4A. Fluorescence spectra of Cy7@GluAC4A (10/12 µm) before and after the reduction by SDT (10 mm) in PBS (10 mm, pH 7.4) at 37 °C. J) Fluorescent responses of Cy7@GluAC4A (0.10/0.10 mm) upon exposure to major blood components. The inset displays fluorescence images of free Cy7 (0.10 mm), Cy7@GluAC4A ((0.10/0.10 mm), and Cy7@GluAC4A ((0.10/0.10 mm) + NADPH (50 µM) + DT‐diaphorase (0.40 µm) in mouse serum.

Briefly, GluAC4A was synthesized as follows. By directly reacting calix[4]arene with p‐carboxybenzenediazonium chloride at 2 °C for 3 h, CAC4A, which can be linked with propargyl amine to obtain CAC4A‐Alk by a condensation reaction, was obtained in quantitative yield. Next, CAC4A‐Alk conjugated with acetyl glucose (GluAC4A‐Ac) was obtained via a click reaction of CAC4A‐Alk with Glu‐N_3_. Finally, the targeted compound GluAC4A was produced by the deprotection of the acetyl group (**Figure** **3**A).^[^ ^21^ ^]^

GluAC4A is an amphiphilic compound, as evidenced by its critical aggregation concentration of 8 µm (Figure S4, Supporting Information). The self‐assembly of GluAC4A led to the formation of nanoparticles with a hydrated diameter of 43 nm as determined by dynamic light scattering (DLS) measurements (Figure 3B). The zeta potential indicated that the assembly carried a negative charge of −11.27 mV (Figure 3C). Transmission electron microscopy (TEM) images, of GluAC4A, together with the DLS results, further illustrate its spherical morphology with a consistent size (Figure S5, Supporting Information).

The binding affinity of GluAC4A for Lip was studied using a phase‐solubility diagram. GluAC4A and Lip were combined in a 1:1 ratio by grinding and were subsequently dissolved in phosphate‐buffered saline (PBS) (Figure S6, Supporting Information). The maximum dissolution concentration of Lip@GluAC4A was 3.10 mm, indicating that the solubility increased 696‐fold compared with that of free Lip (Figure S7, Supporting Information). According to the phase solubility diagram (Figure 3D), the binding affinity between GluAC4A and Lip was 3.50 ± 0.49 × 10^6^ M^−1^ (Table S2, Supporting Information). The high binding affinity observed between Lip and GluAC4A indicates that the temporal and spatial consistency of Lip and GluAC4A can be maintained until reaching the BBB. Upon the loading of Lip, the size distribution, morphology, and surface potential of the Lip@GluAC4A nanoparticles remained almost unchanged compared with those of free GluAC4A, which was verified by DLS, zeta potential measurements, and TEM (Figure 3B,C; Figure S5, Supporting Information). Importantly, the Lip@GluAC4A nanoparticles exhibited stability over a 7‐day continuous testing period, with no significant alterations in size (Figure S8, Supporting Information).

To investigate the responsiveness of GluAC4A to hypoxia, we added sodium dithionite (SDT), which is a chemical mimic of azoreductase and continuously monitored the absorbance at 420 nm using an ultraviolet‐visible spectrometer. Upon SDT addition, the characteristic yellow color of GluAC4A disappeared, indicating complete reduction, that is, all four azo groups of GluAC4A were reduced (Figure 3E). The reduction kinetics were quantified by real‐time monitoring of the absorbance at 420 nm (Figure 3F). The absorbance attenuation curve was accurately fitted using a quasi‐first‐order reaction decay model (adjusted *R* ^2^ > 0.995), yielding a rate constant of 0.94 min^−1^ and a half‐life of 0.74 min (Figure S9, Supporting Information). To explore hypoxia‐induced drug release, we employed the fluorescence indicator 1,1′,3,3,3′,3′‐hexamethylindotricarbocyanine iodide (Cy7) as a model drug. The binding affinity of Cy7 with GluAC4A was 1.5 ± 0.20 × 10⁸ M^−1^, as measured by fluorescence titration (Figure 3G). The fluorescence of Cy7 recovered upon the addition of SDT to Cy7@GluAC4A, indicating the release of Cy7 (Figure 3H). Release kinetics were studied by real‐time monitoring of fluorescence. The fluorescence intensity of Cy7 gradually recovered in a time‐dependent manner, reaching saturation 5 min after the addition of SDT to the Cy7@GluAC4A complex, yielding a rate constant of 3.30 min^−1^ and a half‐life of 0.21 min (Figure 3I; Figure S10, Supporting Information). Moreover, we investigated the nonspecific leakage of Cy7. The fluorescence of Cy7@GluAC4A did not increase upon exposure to various blood components, highlighting its exceptional stability in physiological environments (Figure 3J). In addition, we examined the fluorescence intensity of Cy7@GluAC4A in mouse serum (Figure 3J, inset). Compared with free Cy7, Cy7@GluAC4A displayed almost no appreciable emission in mouse serum, demonstrating its resistance to interference from biological components. Upon the addition of DT‐diaphorase, an azoreductase, the fluorescence of Cy7@GluAC4A markedly increased, and the intensity was comparable with that of free Cy7.

### 2.4 GluAC4A Enhances the Penetration of Lip into the Ischemic Brain Parenchyma In Vivo and Exhibits Hypoxia Responsiveness

To investigate the ability of GluAC4A to penetrate the BBB into the brain parenchyma and achieve responsive release in ischemic brain tissues in vivo, we used Cy7 as a fluorescent probe and incorporated it into GluAC4A to form Cy7@GluAC4A. Both Cy7@GluAC4A and free Cy7 were administered intravenously to MCAO mice. In vivo fluorescence imaging was performed at 3, 6, 12, and 24 h post‐injection. Additionally, we assessed the *ex vivo* biodistribution of GluAC4A at 3, 12, and 24 h post‐injection.

**Figure 4:**
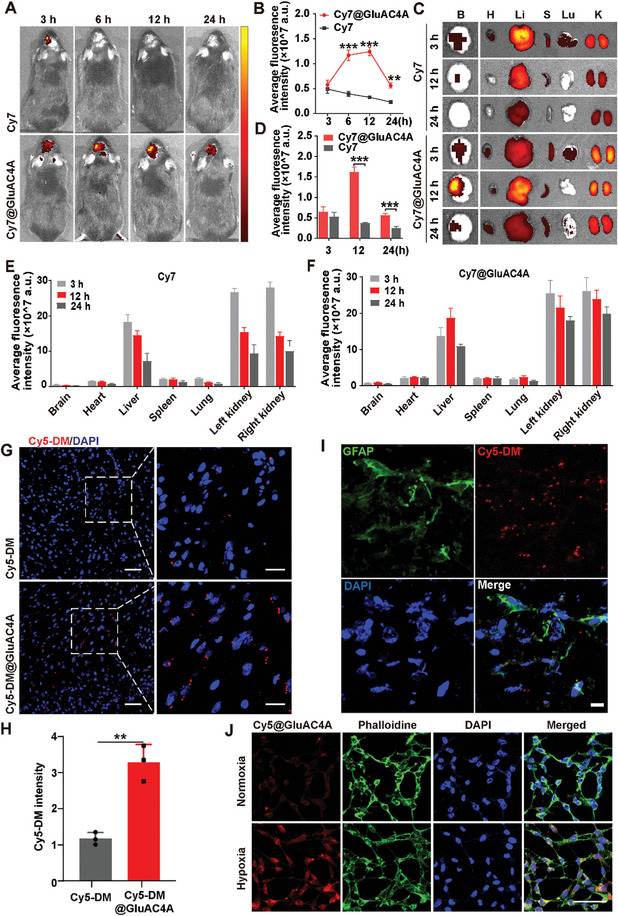
GluAC4A enhances the penetration of the Lip into the ischemic brain parenchyma in vivo and exhibits hypoxia responsiveness. A,B) In vivo fluorescence images and quantification of mean fluorescence intensities in rtPA‐treated MCAO mice 3, 6, 12, and 24 h after tail vein injections of Cy7 and Cy7@GluAC4A (*n* = 3/group). C,D) Ex vivo fluorescence images of isolated mouse organs and mean fluorescence intensities 3, 12, and 24 h after tail vein injections of Cy7 and Cy7@GluAC4A (*n* = 3/group). (B: brain, H: heart, Li: liver, S: spleen, Lu: lung, K: kidneys). E,F) Quantification of fluorescence intensity in vital organs of rtPA‐treated MCAO mice 3, 12, and 24 h after injections of Cy7 and Cy7@GluAC4A. G) Fluorescence images acquired by CLSM demonstrating the accumulation of Cy5‐DM in ischemic brain tissues 12 h after injections of Cy5‐DM and Cy5‐DM@GluAC4A. Scale bars: 50 µm. H) Quantification of relative fluorescence intensities of Cy5‐DM in the Cy5‐DM and Cy5‐DM@GluAC4A groups (*n* = 3/group). I) Representative confocal images showing the uptake of Cy5‐DM@GluAC4A by astrocytes in the ischemic ipsilateral hemisphere 12 h after the Cy5‐DM@GluAC4A injection. Scale bars: 10 µm. J) Fluorescence images of astrocytes treated with Cy5‐DM@GluAC4A under normoxic and hypoxic conditions, followed by staining with phalloidin and DAPI. Data are presented as the mean ± SD, ^*^ *p* < 0.05, ^**^ *p* < 0.01, ^***^ *p* < 0.001.

We monitored changes in the fluorescence of Cy7 in vivo in the brains of MCAO mice over time (**Figure** **4**A). At 3 h post‐injection, no significant differences were observed between the Cy7 and Cy7@GluAC4A groups. However, the fluorescence intensity gradually decreased in the Cy7 group, whereas in the Cy7@GluAC4A group, a distinct pattern was observed: fluorescence increased progressively, peaked at 12 h, and then declined by 24 h. Notably, Cy7@GluAC4A displayed significantly higher fluorescence at 6, 12, and 24 h than Cy7 alone (Figure 4B). This suggests that compared with free Cy7, Cy7@GluAC4A has a higher ability to cross the BBB into the brain parenchyma and Cy7 can be released from GluAC4A in the ischemic brain region. These results demonstrate the capability of GluAC4A to release the drug and simultaneously turn on fluorescence in a hypoxic microenvironment in vivo.

The ex vivo fluorescence of Cy7 in the brain tissue was detectable at 3 h, significantly diminished by 12 h, and barely detectable by 24 h (Figure 4C). Conversely, Cy7 fluorescence in Cy7@GluAC4A‐treated brains demonstrated a remarkable surge at 12 h post‐injection, consistent with the in vivo imaging results. A quantitative analysis (Figure 4D) demonstrated significantly higher fluorescence intensity in the ischemic hemisphere of the Cy7@GluAC4A‐treated group than in the Cy7‐treated group at 12 and 24 h post‐injection. The assessment of vital organs showed that the fluorescence was mainly accumulated in the liver and kidneys compared with other tissues, which could be attributed to Cy7 undergoing metabolism primarily in the liver and kidneys (Figure 4C,E,F).

As Cy7 is not easily excitable under confocal microscopy, we also used 1,1′,3,3,3′,3′‐hexamethylindodicarbocyanine (Cy5‐DM) as an imaging probe to further investigate the fate and biodistribution of GluAC4A using confocal microscopy. The binding constant of Cy5‐DM with GluAC4A was measured as *K*_a_ = 2.5 ± 0.74 × 10^8^ M^−1^ (Figure S11, Supporting Information). Cy5‐DM and Cy5‐DM@GluAC4A were administered via tail vein injection 12 h post‐MCAO. Brain tissues were collected and sectioned for microscopy 12 h post‐injection. Cy5‐DM showed abundant accumulation in the ischemic brain regions of MCAO mice in the Cy5‐DM@GluAC4A group, whereas minimal accumulation was observed in the Cy5‐DM group (Figure 4G,H). Our results indicated that Cy5‐DM released from GluAC4A accumulated in ischemic brain tissues. Since we observed that ferroptosis occurred mainly in astrocytes in the MCAO mouse model after rtPA treatment (Figure 1G–J), the co‐localization of Cy5‐DM and astrocytes was further examined in ischemic brain regions, which revealed that Cy5‐DM co‐localized with astrocytes (GFAP^+^ cells) (Figure 4I).

To verify cellular uptake and intracellular drug release, intracellular drug localization was studied using confocal microscopy under normoxic and hypoxic conditions. Cy5‐DM@GluAC4A was incubated with astrocytes under normoxic and hypoxic conditions. Remarkable fluorescence recovery of Cy5‐DM was observed in cells under hypoxic rather than normoxic conditions (Figure 4J), indicating the responsiveness of GluAC4A to hypoxia.

Collectively, these results suggest that GluAC4A can penetrate the BBB into the brain parenchyma and release the drugs in ischemic brain tissues, allowing them to perform biological functions.

### 2.5 Evaluating the Toxicity of GluAC4A and Lip@GluAC4A In Vivo and In Vitro

**Figure 5:**
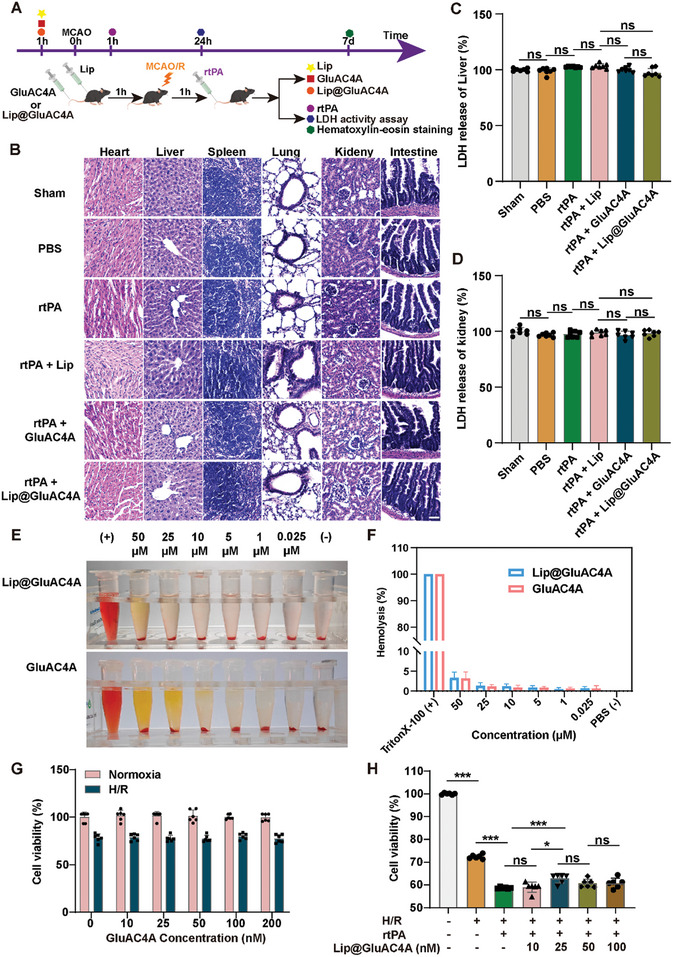
Evaluating the toxicity of GluAC4A and Lip@GluAC4A in vivo and in vitro. A) Experimental timeline. B) Representative H&E staining images of heart, liver, spleen, lung, kidney, and intestine tissue sections of MCAO mice treated with sham or various treatments (n = 3/group). Scale bars, 50 µm. C,D) LDH levels in the liver and kidneys of MCAO mice treated with sham or various treatments (*n* = 7/group). E,F) Hemolysis rate after incubation with rabbit erythrocytes (*n* = 3/group). G) Astrocytes were pre‐treated with GluAC4A at varying concentrations (10, 25, 50, 100, and 200 nm) for 12 h. Cell viability was assessed after exposure to normoxic or hypoxic conditions (*n* = 6/group). H) Cell viability of astrocytes after incubation with different concentrations (10, 25, 50, and 100 nm) of Lip@GluAC4A under hypoxic conditions with rtPA treatment (*n* = 6/group).

We conducted gross histological analyses of the vital organs of mice subjected to various treatments to assess the systemic toxicity of GluAC4A in vivo; the administration and duration of treatment in mice are shown in **Figure** **5**A. Hematoxylin and eosin (H&E) staining of tissue sections did not reveal any pathological changes in the heart, liver, spleen, lungs, kidneys, or intestines of mice treated with Lip, GluAC4A, or Lip@GluAC4A (Figure 5B). These organs appeared structurally intact, with cells displaying an orderly arrangement, eosinophilic cytoplasm, and round nuclei. The nuclei were centrally located and exhibited clear morphology with no evidence of cell necrosis. The absence of pathological changes in the vital organs of the mice treated with Lip@GluAC4A supports the safety profile of this drug delivery system. This aspect is of paramount importance considering the translation of this research into clinical applications where patient safety is the top priority. Since GluAC4A is mainly metabolized by the liver and kidneys, we used a lactate dehydrogenase (LDH) release assay to detect changes in LDH levels in the liver and kidneys of MCAO mice after the injection of Lip@GluAC4A and observed that GluAC4A did not cause liver or kidney injury (Figure 5C,D). A plasma hemolysis assay was performed to investigate the in vivo biocompatibility of Lip@GluAC4A and GluAC4A. Our results showed that Lip@GluAC4A and GluAC4A displayed hemolysis ratios much lower than the internationally recognized standard (5%) (Figure 5E,F).

Next, we explored the cytotoxicity of the supramolecular carrier GluAC4A and the supramolecular prodrug Lip@GluAC4A under normoxic and hypoxic conditions in vitro. We assessed the cytotoxicity of GluAC4A over a concentration range of 10.0−200 nM using a CCK8 assay. After a 24 h incubation period under normoxic conditions, astrocytes exhibited a survival rate of ≈100%. Moreover, under hypoxic conditions, varying concentrations of GluAC4A had no significant effect on cell survival when the impact of hypoxia on cell viability was taken into account, indicating negligible cytotoxicity (Figure 5G).

Subsequently, we measured the viability of astrocytes after treatment with different concentrations (10.0−100 nM) of Lip@GluAC4A in vitro. Lip@GluAC4A improved the survival rate of astrocytes at a concentration of 25.0 nm, with higher concentrations not significantly enhancing survival (Figure 5H). These findings are consistent with the effect of Lip concentration on astrocyte survival under hypoxic conditions (data not shown). This suggests that Lip@GluAC4A enhanced cellular uptake and improved cell viability without altering the effective drug‐loading concentration.

In summary, we did not observe any toxic effects of GluAC4A in distant organs or astrocytes, indicating the safety and feasibility of this therapeutic strategy.

### 2.6 Lip@GluAC4A Attenuates rtPA‐Induced Side Effects in Mice

**Figure 6:**
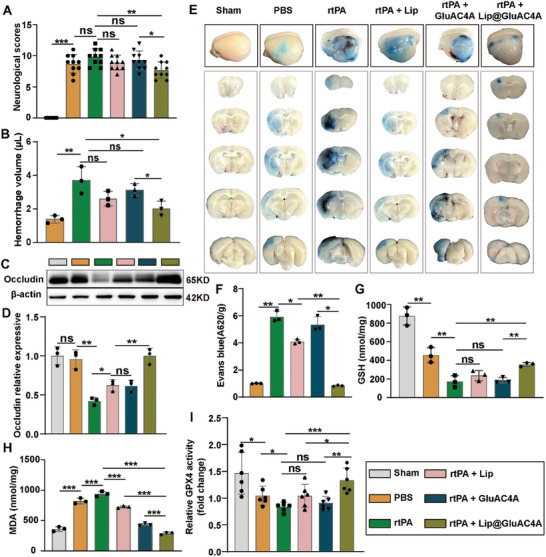
Lip@GluAC4A Attenuates rtPA‐induced Side Effects in Mice. A) The mNSS test (*n* = 10/group). B) Quantification of cerebral hemorrhage using the Drabkin reagent (*n* = 3/group). C,D) Western blot images and quantification of occludin expression in MCAO mice brain (*n* = 3/group). E,F) Representative images and quantification of Evans blue extravasation 24 h after MCAO in mice. G–I) GSH (G, *n* = 3/group), MDA (H, *n* = 3/group), and GPX4 activity (I, *n* = 6/group) levels of the ischemic brains 24 h after MCAO in mice (*n* = 3/group). Data represent the mean ± SD, ^*^ *p* < 0.05, ^**^ *p* < 0.01, ^***^ *p* < 0.001, ^****^ *p* < 0.0001.

To further demonstrate the application of the supramolecular carrier GluAC4A in attenuating rtPA‐induced side effects in the MCAO mouse model, we investigated the antiferroptotic effect of Lip@GluAC4A and its impact on BBB integrity and the prognosis of rtPA‐treated MCAO mice. Our findings revealed that neurological deficits, as measured by the modified neurological severity score (**Figure** **6**A),^[^ ^3^, ^35^ ^]^ and brain hemorrhage (Figure 6B), were significantly reduced after treatment with rtPA + Lip@GluAC4A compared with treatment with only rtPA. However, no such effect was observed in the rtPA + Lip and rtPA + GluAC4A groups. Expression of the tight junction protein occludin (Figure 6C,D) and BBB leakage, as indicated by Evans blue extravasation (Figure 6E,F), remained unchanged after treatment with rtPA + GluAC4A and improved to a certain extent after treatment with rtPA + Lip but remarkably improved after treatment with rtPA + Lip@GluAC4A, indicating protective effect of Lip@GluAC4A on the BBB in vivo.

Furthermore, rtPA + Lip@GluAC4A treatment significantly enhanced the antioxidant effect, as indicated by elevated GSH levels, compared with rtPA‐only and rtPA + GluAC4A treatments (Figure 6G). Moreover, lipid peroxidation, as indicated by reduced MDA levels (Figure 6H), was significantly decreased in the rtPA + Lip@GluAC4A treatment group than in the rtPA‐only, rtPA + GluAC4A, and rtPA + Lip treatment groups. rtPA + Lip and rtPA + GluAC4A treatments reduced the MDA level but did not increase the GSH level compared with rtPA‐only treatment. Additionally, we evaluated the effects of Lip and Lip@GluAC4A on GPX4 activity. We observed that Lip did not affect GPX4 activity in vivo. However, Lip@GluAC4A enhanced GPX4 activity (Figure 6I). These results indicate that the delivery of Lip by GluAC4A enhances Lip accumulation in the ischemic brain tissue, thereby improving its antiferroptotic effect, reducing BBB disruption, and consequently decreasing rtPA‐associated brain hemorrhage, ultimately improving neurological function. This improvement can be attributed to the GluAC4A optimizing in vivo distribution and enhancing Lip accumulation in the ischemic brain region.

This novel approach for mitigating the side effects of rtPA treatment is promising for enhancing stroke care. It not only addresses the detrimental consequences of rtPA treatment but also helps maintain the integrity of the BBB, which is crucial for preventing complications such as hemorrhagic transformation. Administering Lip@GluAC4A before MCAO may limit the immediate clinical relevance in patients experiencing first‐episode acute ischemic stroke. Our findings suggest that the prophylactic use of Lip@GluAC4A in high‐risk patients with recurrent ischemic stroke is promising. This strategic consideration may extend the applicability of hypoxia‐responsive Lip@GluAC4A to ischemic stroke.

### 2.7 The Effect of SLC7A11 and GPX4 Activity on the Protective Effects of Lip@GluAC4A Treatment on the BBB

**Figure 7:**
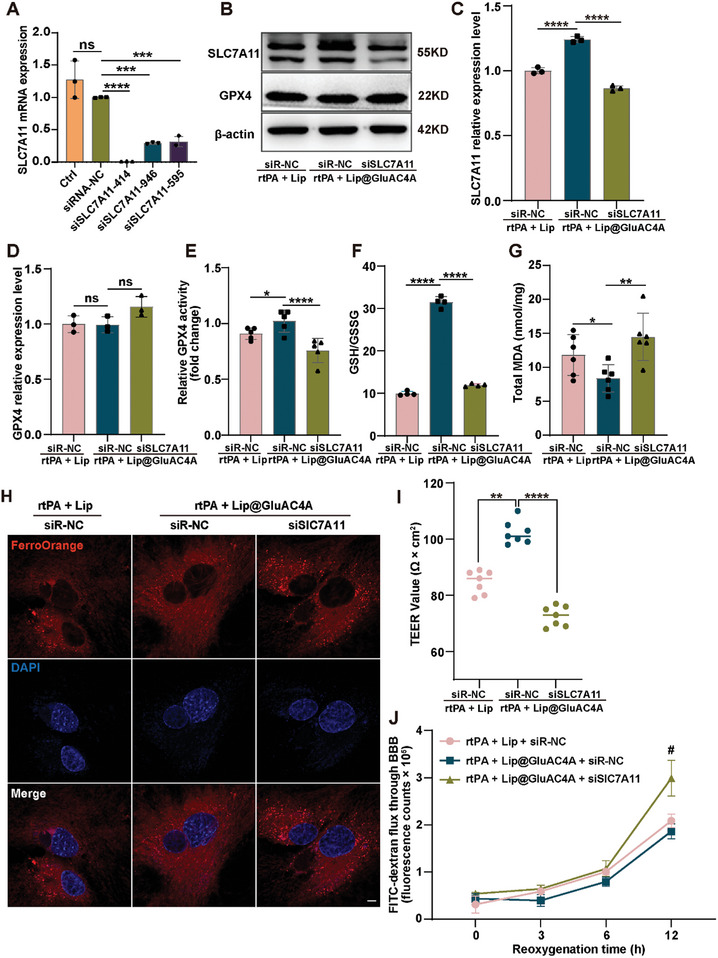
The Regulation of SLC7A11 on GPX4 Activity Involved in the Protective Effects of Lip@GluAC4A Treatment on the BBB. A) RT‒qPCR was used to measure the mRNA level of SLC7A11 following SLC7A11 knockdown (*n* = 3/group); B–D) Western blot images and quantification of SLC7A11 and GPX4 following SLC7A11 knockdown (*n* = 3/group); E–G) GPX4 activity (E, *n* = 5/group), the ratio of GSH/GSSG (F, *n* = 4/group), and the total MDA level (G, *n* = 6/group) were measured. H) Representative confocal images of Fe^2+^ in astrocytes following SLC7A11 knockdown (*n* = 4/group). Scale bars: 5 µm. I,J) TEER (I, *n* = 7/group) and FITC‐dextran permeability (J, *n* = 6/group) were measured. ^#^, Lip@GluAC4A + siR‐NC versus Lip@GluAC4A + siSLC7A11, *p* < 0.05. Data represent the mean ± SD, ^*^ *p* < 0.05, ^**^ *p* < 0.01, ^***^ *p* < 0.001, ^****^ *p* < 0.0001.

We conducted a series of in vitro experiments to further investigate the mechanism by which Lip@GluAC4A maintains BBB integrity via the SLC7A11/GPX4 pathway. The SLC7A11/GPX4 pathway plays a pivotal role in ferroptosis; however, its involvement in protecting against BBB disruption remains unclear. First, we knocked down SLC7A11 in astrocytes using an siRNA against it (siSLC7A11), which resulted in a significant decrease in SLC7A11 mRNA and protein levels compared with that in astrocytes treated with the scrambled control (siR‐NC) (**Figure** **7**A–C). These results indicated that siSLC7A11 could effectively interfere with the expression of SLC7A11 mRNA. However, although siSLC7A11 treatment did not induce any changes in GPX4 protein expression (Figure 7B,D), it significantly reduced GPX4 activity compared with the siR‐NC treatment (Figure 7E). We also observed a reduced GSH/GSSG ratio in siSLC7A11‐treated astrocytes compared with that in the siR‐NC‐treated astrocytes (Figure 7F). Our study revealed increased SLC7A11 expression and sustained GPX4 levels, accompanied by increased GPX4 activity and elevated GSH levels in astrocytes following Lip@GluAC4A application. Previous studies have indicated that Lip alleviates ferroptosis by promoting the expression of SLC7A11 and GPX4.^[^ ^36^ ^]^ In addition, we examined other ferroptosis pathways such as the level of Fe^2+^ in the iron metabolic pathway and the level of MDA, an indicator of lipid peroxidation. We observed that the levels of Fe^2+^ and MDA increased after siSLC7A11 treatment (Figure 7G,H), suggesting that the knockdown of SLC7A11 not only affected amino acid metabolism but also influenced other pathways related to ferroptosis and jointly promoted ferroptosis. Finally, we constructed an in vitro BBB model and observed that TEER decreased while FITC‐dextran permeability increased in siSLC7A11‐treated astrocytes compared with siR‐NC‐treated astrocytes (Figure 7I,J), suggesting that SLC7A11 exerted protective effects on the BBB.

## 3 Conclusion 

Herein, we discovered a novel mechanism by which rtPA induces hemorrhagic transformation in ischemic stroke by triggering ferroptosis in astrocytes. Our results showed that the ferroptosis inhibitor Lip effectively reduced astrocyte death and maintained BBB integrity. Therefore, we proposed a therapeutic strategy using Lip to alleviate rtPA‐induced side effects in the treatment of acute ischemic stroke. We successfully designed and synthesized a macrocyclic carrier, GluAC4A, that could efficiently deliver and release Lip at the lesion site. The deep cavity of GluAC4A enabled it to bind strongly with Lip, which improved its solubility and prevented off‐target leakage, enabling Lip@GluAC4A to target the BBB. Additionally, the responsiveness of azo groups to hypoxia enabled the controlled release of Lip in the lesion location. Lip@GluAC4A significantly reduced astrocyte death, protected the BBB, and alleviated hemorrhagic transformation by maintaining the SLC7A11/GPX4 signaling pathway, demonstrating the theoretical basis and therapeutic target of Lip@GluAC4A. Consequently, this study not only offers new insights into the side effects of rtPA treatment but also presents a novel therapeutic strategy for the effective mitigation of such side effects, which is of great significance for the treatment and prognosis of acute ischemic stroke.

## Ethical Statement

All animal procedures were evaluated and authorized by the Laboratory Animal Center of Tianjin Huanhu Hospital. The approval numbers of the animal experiments were as follows: HHLL‐20210006. All animal experiments were performed in accordance with the guidelines outlined in the National Institutes of Health guide for the care and use of laboratory animals (NIH Publications No. 8023, revised 1978).

## Conflict of Interest

The authors declare no conflict of interest.

## Supporting information

Supporting Information

## Data Availability

Research data are not shared.
