# Supplementary material for: Alleviating Recombinant Tissue Plasminogen Activator‐induced Hemorrhagic Transformation in Ischemic Stroke via Targeted Delivery of a Ferroptosis Inhibitor
Source: Adv Sci (Weinh). 2024 Apr 22;11(24):2309517. doi: 10.1002/advs.202309517 (PMC11199968; doi:10.1002/advs.202309517)
Supplement: Supplementary file 1 — Supporting Information [file ADVS-11-2309517-s001.pdf]

ADVANCED SCIENCE
Open Access

Supporting Information

for *Adv. Sci.*, DOI 10.1002/advs.202309517

Alleviating Recombinant Tissue Plasminogen Activator-induced Hemorrhagic Transformation in Ischemic Stroke via Targeted Delivery of a Ferroptosis Inhibitor

Yan-Qin Geng, Li-Na Qiu, Yuan-Qiu Cheng, Juan-Juan Li, Yi-Lin Ma, Cheng-Cheng Zhao, Ying Cai, Xue-Bin Zhang, Jieli Chen, Yu-Chen Pan, Ke-Rang Wang, Xiu-Hua Yao*, Dong-Sheng Guo* and Jia-Ling Wu**

Alleviating Recombinant Tissue Plasminogen Activator-induced Hemorrhagic Transformation in Ischemic Stroke via Targeted Delivery of a Ferroptosis Inhibitor

Yan-Qin Geng[#], Li-Na Qiu[#], Yuan-Qiu Cheng[#], Juan-Juan Li, Yi-Lin Ma, Cheng-Cheng Zhao, Ying Cai, Xue-Bin Zhang, Jieli Chen, Yu-Chen Pan, Ke-Rang Wang, Xiu-Hua Yao*, Dong-Sheng Guo*, Jia-Ling Wu**

Y.-Q. Geng, J.-L. Wu
School of Medicine
Nankai University
Tianjin 300071, China
TianJin Huanhu Hospital
Tianjin 300350, China
E-mail: wywjl2009@hotmail.com (J.-L. Wu)
L.-N. Qiu, J.-L. Wu
Department of Neurology
Tianjin Huanhu Hospital
Tianjin 300350, China
L.-N. Qiu, Y. Cai, J.-L. Chen, X.-H. Yao, J.-L. Wu
Tianjin Key Laboratory of Cerebral Vascular and Neurodegenerative Diseases
Tianjin Neurosurgical Institute
Tianjin Huanhu Hospital
Tianjin 300350, China
E-mail: yao_xiuhua87@163.com (X.-H. Yao)
Y.-Q. Cheng, Y.-C. Pan, D.-S. Guo
College of Chemistry
State Key Laboratory of Elemento-Organic Chemistry
Key Laboratory of Functional Polymer Materials (Ministry of Education)
Frontiers Science Center for New Organic Matter
Collaborative Innovation Center of Chemical Science and Engineering (Tianjin)
Nankai University
Tianjin 300071, China
E-mail: dshguo@nankai.edu.cn (D.-S. Guo)
J.-J. Li, K.-R. Wang

College of Chemistry and Environmental Science
Key Laboratory of Medicinal Chemistry and Molecular Diagnosis (Ministry of Education)
Key Laboratory of Chemical Biology of Hebei Province
Hebei University
Baoding 071002, China
E-mail: kerangwang@hbu.edu.cn (K.-R. Wang)
Y.-L. Ma, C.-C. Zhao
Clinical College of Neurology
Neurosurgery and Neurorehabilitation
Tianjin Medical University
Tianjin 300071, China
X.-B. Zhang
Department of Pathology
Tianjin Huanhu Hospital
Tianjin 300350, China
D.-S. Guo
Xinjiang Key Laboratory of Novel Functional Materials Chemistry
College of Chemistry and Environmental Sciences
Kashi University
Kashi 844000, China

Table of Content

1. Materials. **1**

2. Samples. **1**

3. Animals. **1**

4. Instruments. **2**

5. Methods. **2**

5.1 Synthesis of GluAC4A. **2**

5.1.1 Synthesis of CAC4A. **2**

5.1.2 Synthesis of GluAC4A. **3**

5.2 Solubility measurement of Lip. **6**

5.3 Reduction kinetics of GluAC4A. **6**

5.4 Release kinetics of Cy7@GluAC4A. **6**

5.5 Phase-solubility curve. **7**

5.6 TEM measurements. **7**

5.7 Fluorescence titrations. **7**

5.8 Fluorescence responses of Cy7@GluAC4A and Cy5-DM@GluAC4A upon the addition of various biologically coexisting species. **8**

5.9 Mouse model of ischemic stroke. **8**

5.10 Laser speckle imaging **8**

5.11 TTC staining **9**

5.12 Assessing GluAC4A biodistribution through *in vivo* imaging. **9**

5.13 Laser confocal imaging. **9**

5.14 Experimental treatment groups *in vivo*. **10**

5.15 Neurological behavioral score assessment. **10**

5.16 Evaluating systemic toxicity of GluAC4A and Lip@GluAC4A after prolonged *in vivo* accumulation. **11**

5.17 Lactate dehydrogenase (LDH) release detection **11**

5.18 Hemolysis assay. 11

5.19 Quantification of cerebral hemorrhage. 11

5.20 BBB disruption measurement. 11

5.21 Double immunostaining. 12

5.22 Cell culture and hypoxia/reoxygenation experiments. 12

5.23 *In vitro* astrocyte culture experimental groups. 12

5.24 Intracellular uptake of Cy5-DM@GluAC4A and hypoxia-induced response. ... 13

5.25 *In vitro* cytotoxicity assessment of GluAC4A and Lip@GluAC4A. 13

5.26 Assessment of lipid peroxidation using BODIPY and flow cytometry. 13

5.27 *In vitro* BBB model. 13

5.28 TEER and permeability assessment. 14

5.29 Imaging with FerroOrange. 14

5.30 Imaging of Lipid ROS. 14

5.31 GSH and MDA assay. 14

5.32 GPX4 activity assessment. 15

5.33 SLC7A11 knockdown in astrocytes. 15

5.34 Western blotting. 16

5.35 RT-qPCR. 16

5.36 Statistical analysis. 16

6. Supporting results. 17

6.1 The expression of SLC7A11 and GPX4 on neurons. 17

6.2 The expression of PTGS2 mRNA *in vivo* and *in vitro* models. 17

6.3 The expression of COX2 in astrocytes. 17

6.4 Detection the critical aggregation concentration (CAC) of GluAC4A. 18

6.5 Characterization of the Lip@GluAC4A co-assembly. 18

6.6 GluAC4A improves the solubility of Lip in PBS. 19

6.7 Phase-solubility curve. 19

6.8 Stability testing of Lip@GluAC4A. **19**

6.9 Reduction kinetics of GluAC4A. **20**

6.10 Release kinetics of Cy7@GluAC4A. **20**

6.11 Binding affinities of Cy5-DM with GluAC4A. **21**

7. References **22**

1. Materials.

All the reagents and solvents were commercially available and used as received unless otherwise specified purification. Sodium hyposulfite (SDT) was purchased from J&K Chemical. 4-Aminobenzoic acid. 1,1',3,3,3',3'-hexamethylindotricarbocyanine iodide (Cy7) was purchased from Sigma-Aldrich. 1,1',3,3,3',3'-Hexamethylindodicarbocyanine (Cy5-DM) was purchased from OKeanos Tech. Co., Ltd. 2-(7-Azabenzotriazol-1-yl)-N,N,N',N'-tetramethyluronium hexafluorophosphate (HATU), N,N-diisopropylethyl-amine (DIPEA), propargulamine, copper sulfate pentahydrate ($CuSO_4 \cdot 5H_2O$), sodium ascorbate and sodium methanolate (CH_3NaO) were purchased from TCI. 5,11,17,23-Tetrakis[(p-carboxy-phenyl)azo]-25,26,27,28-tetra-hydroxy calix[4]arene (CAC4A) was synthesized according to the previous literature. Cell counting kit-8 (CCK-8) was purchased from Beyotime. Dulbecco's modified Eagle's medium (DMEM), 1% penicillin–streptomycin (PS), fetal bovine serum (FBS), 0.25% trypsin-EDTA were purchased from Gibco (USA). Triton X-100, 4% paraformaldehyde (PFA), PBS and DAPI were purchased from Solarbio (China). rtPA (Alteplase) was obtained from Boehringer Ingelheim (Germany). Anti-GPX4 antibodies were purchased from Abcam (England). Anti-GFAP and anti-NeuN antibodies were obtained from Cell Signaling Technology (USA). Anti-SLC7A11 antibody was purchased from Santa Cruz (CA). Anti-COX2 antibody was purchased from proteintech (China), Anti- S100β antibody was purchased from proteintech (China), Liproxstatin-1 (Lip) was purchased from Selleck (S7699, USA).

2. Samples.

The phosphate buffered saline (PBS) solution of pH = 7.4 was prepared by dissolving 0.60 g of sodium phosphate monobasic dehydrate, 0.87 g disodium phosphate, 8 g sodium chloride and 0.20 g potassium chloride in approximate 900 mL double-distilled water. Titrate to pH = 7.4 at the lab temperature of 25 °C with NaOH and make up volume to 1000 mL with double-distilled water. The pH value of the buffer solution was then verified on a pH-meter calibrated with three standard buffer solutions. GluAC4A nanoparticles were prepared by dissolving the solids in PBS, and it would self-assemble to form assemblies. The sample for transmission electron microscopy (TEM) measurement was prepared by dropping the solution onto a copper grid without staining. The grid was then air-dried.

3. Animals.

Young adult C57BL/6J male mice (8-10 weeks old) were supplied from Beijing Vital River Laboratory Animal Technology. All experiments were conducted in accordance with the Institutional Animal Care and Use Committee guidelines, with random group assignments.

4. Instruments.

^{1}H NMR data were recorded on a Bruker AV400 spectrometer. UV-Vis spectra were recorded in a quartz cell (light path 10 mm) on a Cary 100 UV-Vis spectrophotometer equipped with a Cary dual-cell peltier accessory. The HPLC equipment used Alliance 2695 system with UV detection at 270 nm for Lip. Fluorescence measurements were recorded in a conventional quartz cell (light path 10 mm) on Cary Eclipse and PerkinElmer FL6500. The TEM sample was examined by a TEM (HITACHI HT7700 Exalens). The sample solutions for dynamic light scattering (DLS) measurements were examined on a laser light scattering spectrometer (NanoBrook 173plus and Brookhaven ZetaPals/BI-200SM). Fluorescence images were captured using a confocal laser scanning microscope (CLSM 800, Zeiss, Germany), followed by analysis using ImageJ software. Cell viability was determined by measuring the absorbance of each well at 450 nm using a multimode microplate reader (SpectraMax 190, Molecular Devices, USA).

5. Methods.

5.1 Synthesis of GluAC4A.

5.1.1 Synthesis of CAC4A.

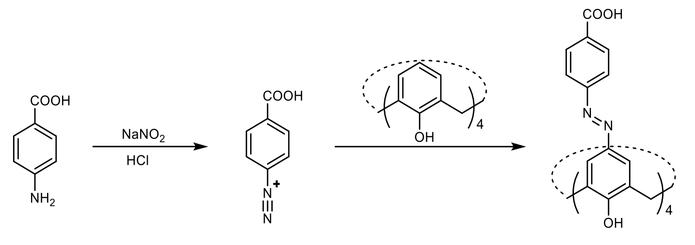

The synthetic route of CAC4A.

CAC4A.[13] 4-aminobenzoic acid (1.37 g, 10 mmol) and concentrated hydrochloric acid (2 mL, 37%) were dissolved in 15 mL water in a round bottom flask. After cooling the solution to 2 °C under an ice-water bath, a solution of sodium nitrite (10 mL, 10 mmol, in water) was slowly added, while the temperature was controlled to lower than 5 °C. The obtained solution was slowly added into a solution of 25,26,27,28-tetrahydroxycalix[4]arene (C4A, 1 g, 2.36 mmol) and sodium acetate (2.46 g, 30 mmol) in MeOH-DMF (26 mL, 5:8, v: v) to obtain a red suspension. After stirring at room temperature for 2 h, hydrochloric acid solution (37%) was added until the solution was adjusted to pH = 1. After warming to 60 °C for 30 min, the mixture was filtered and washed with water and MeOH to obtain a reddish solid of CAC4A in a quantitative yield.

^{1}H NMR (400 MHz, DMSO-d_6, δ) 8.09 (d, 8H, J = 8.0 Hz, Ar-H), 7.88 (d, 8H, J = 8.6 Hz, Ar-

H), 7.88 (s, 8H, calix-Ar-H), 4.49 (s, 4H, Ar-CH2-Ar) ppm. The other bridged hydrogen is obscured by the water peak.

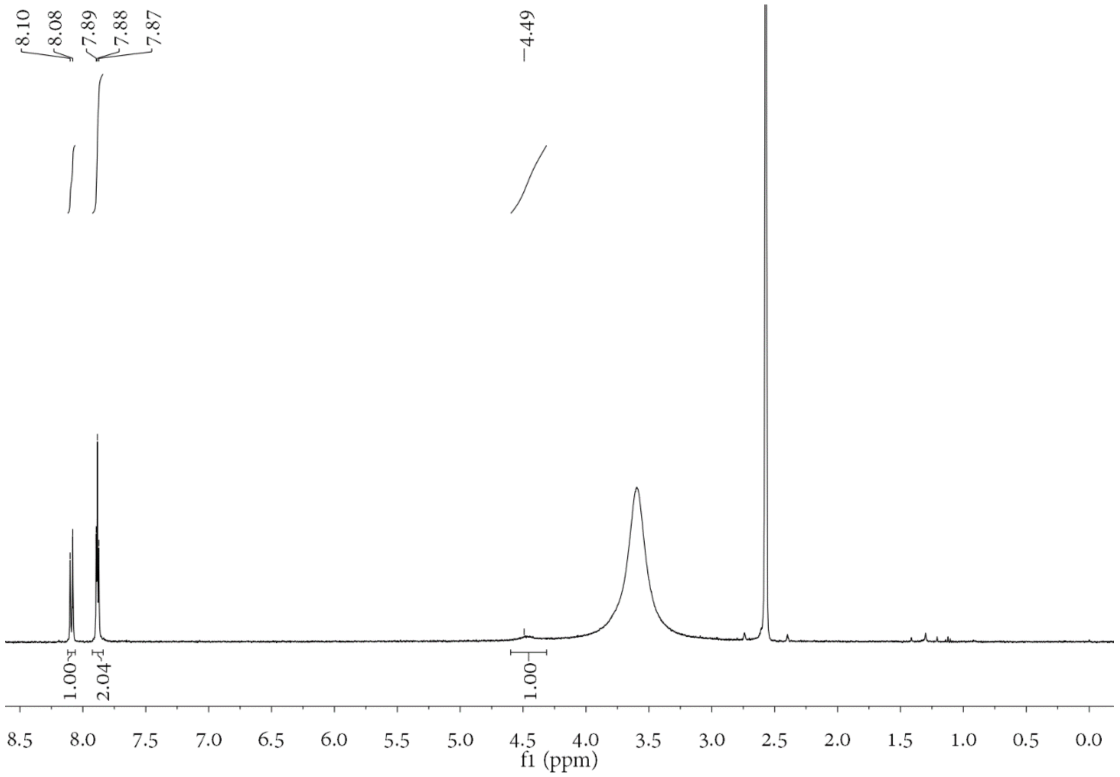

^{1}H NMR spectrum of CAC4A in DMSO-*d*$_6$, 400 MHz, 25 °C.

5.1.2 Synthesis of GluAC4A.

GluAC4A-Ac. Add CAC4A-Alk (200 mg, 0.16 mmol) and Glu-N_3 (320 mg, 0.77 mmol) to 30 mL of THF in a round bottom flask. Then, a water solution of $CuSO_4{\cdot}5H_2O$ (94 mg, 0.38 mmol) and sodium ascorbate (76 mg, 0.38 mmol) was added. The reaction mixture was stirred for 10 h at 55 °C under a N_2 atmosphere. The reaction solvents were evaporated under vacuum. The residue was resolved by CH_2Cl_2 and purified by silica-gel column chromatography using CH_2Cl_2/CH_3OH (v/v, 30/1) as the eluent. GluAC4A-Ac (290 mg) was obtained with a yield of 65%.

^{1}H NMR (400 MHz, DMSO-d_6) δ 9.14 (t, J = 5.2 Hz, 4H, Triazole-N*H*), 7.98 (d, 8H, J = 8.0 Hz, Ar-H), 7.88 (s, 4H, Triazole-H), 7.81 (d, 8H, J = 12.0 Hz, Ar-H), 7.79 (s, 8H, calix-Ar-H), 5.22 (t, J = 8.0 Hz, 4H), 4.91-4.88 (m, 4H), 4.82 (d, 4H, J = 4.0 Hz), 4.74-4.69 (m, 4H), 4.52-4.49 (m, 16H), 4.19-4.15 (m, 4H), 4.10-3.89 (m, 20H), 3.72-3.61 (m, 8H), 2.01 (s, 12H, Ac-H), 1.97 (s, 12H, Ac-H), 1.91 (s, 12H, Ac-H), 1.88 (s, 12H, Ac-H) ppm.

^{13}C NMR (100 MHz, DMSO-d_6) δ 170.54, 170.01, 169.74, 169.43, 165.91, 160.16, 154.32, 145.28, 145.13, 135.23, 131.12, 128.86, 124.44, 123.75, 122.22, 99.60, 72.38, 71.13, 71.01, 68.56, 67.88, 62.12, 54.06, 49.61, 20.97, 20.83, 20.70 ppm.

a

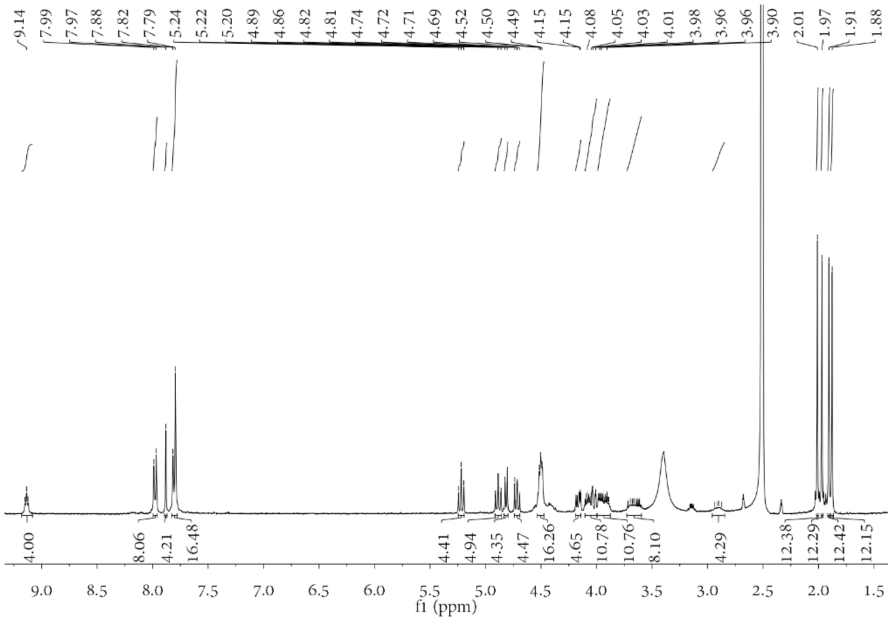

b

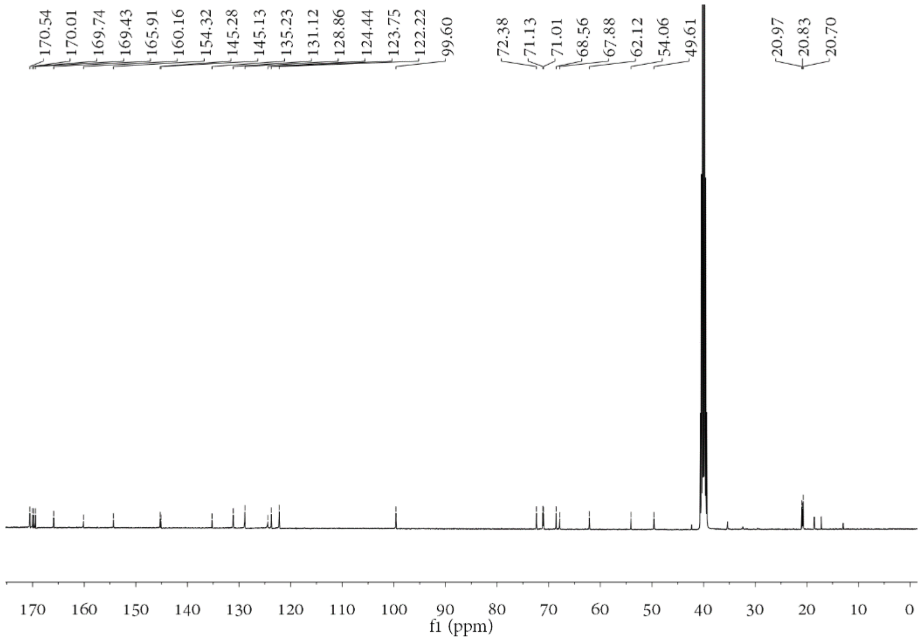

(a) ^{1}H NMR spectrum of GluAC4A-Ac in DMSO-*d*$_6$, 400 MHz, 25 °C, and (b) ^{13}C NMR spectrum of GluAC4A-Ac in DMSO-*d*$_6$, 100 MHz, 25 °C.

GluAC4A.[13b] GluAC4A-Ac (200 mg, 0.0706 mmol) and MeONa (92 mg, 1.68 mmol) were dissolved in anhydrous MeOH (20 mL). The reaction mixture was stirred at room temperature for 10 h. The reaction mixture was placed in a cellulose dialysis tube (cutoff 2000) and dialyzed against water for 2 days. The compound GluAC4A (137 mg) was obtained through lyophilization with a yield of 90%.

^{1}H NMR (400 MHz, DMSO-*d*$_6$) δ 9.11 (t, J = 5.2 Hz, 4H, Triazole-N*H*), 8.04 (s, 4H, Triazole-

H), 7.98 (d, 8H, *J* = 8.0 Hz, Ar-H), 7.81 (d, 8H, *J* = 8.0 Hz, Ar-H), 7.80 (s, 8H, calix-Ar-H), 6.64 (s, 29H, fumaric acid-C*H*=C*H*), 5.06-5.05 (m, 4H), 4.97-4.92 (m, 8H), 4.55-4.50 (m, 20H), 4.22 (d, 4H, *J* = 8.0 Hz), 4.09-4.04 (m, 8H), 3.92-3.87 (m, 8H), 3.68-3.64 (m, 12H) ppm. ^{13}C NMR (100 MHz, DMSO-d_6) δ 166.03, 160.39, 154.31, 145.15, 135.32, 131.14, 128.89, 124.48, 124.21, 122.22, 104.01, 75.84, 73.76, 70.83, 68.61, 67.61, 60.93, 50.08, 35.39 ppm. FTMS (MALDI): [M+Na] $^+$: m/z calcd. for $C_{100}H_{112}N_{24}O_{32}Na^+$: 2183.79, found: 2183.7713.

a

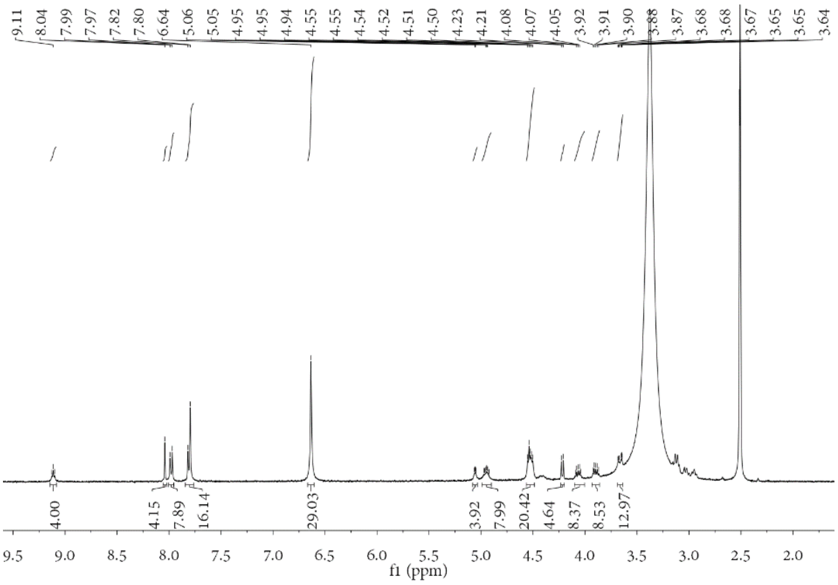

b

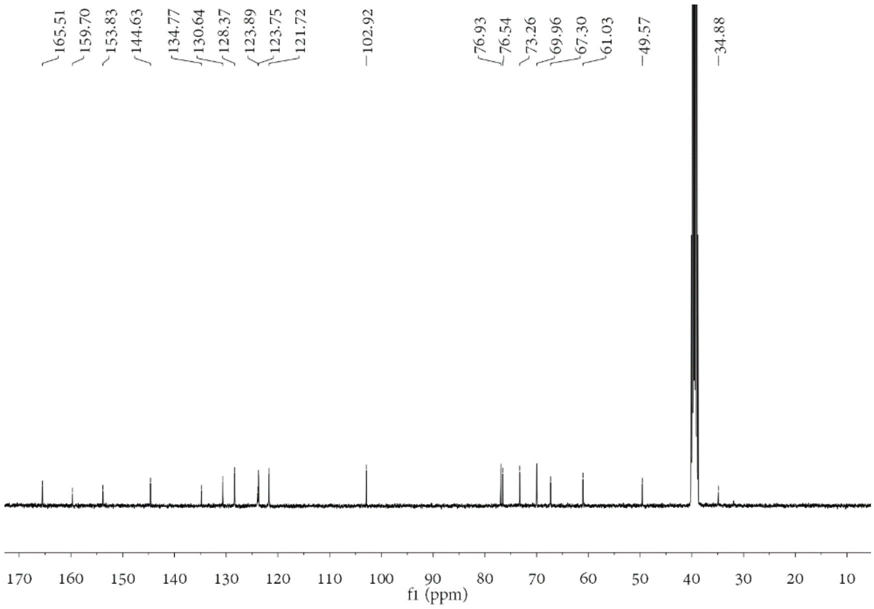

c

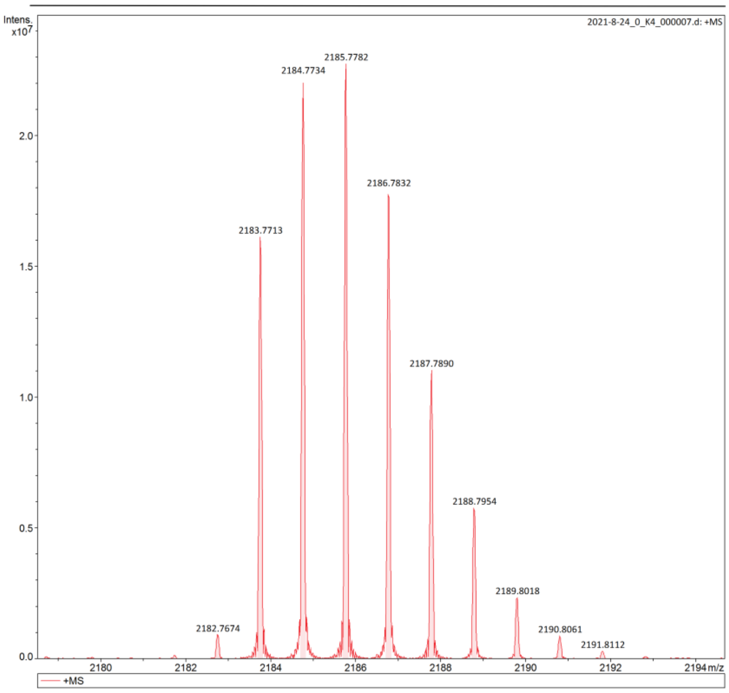

(a) ^{1}H NMR spectrum of GluAC4A in DMSO-*d*$_6$, 400 MHz, 25 °C, (b) ^{13}C NMR spectrum of GluAC4A in DMSO-*d*$_6$, 100 MHz, 25 °C and (c) FTMS of GluAC4A.

5.2 Solubility measurement of Lip.

The solid of Lip or mixture of Lip and GluAC4A was dissolved in PBS (10 mM, pH = 7.4) by shaking at 25 °C overnight, and the insoluble solid was removed by centrifugation at 13400 × g for 5 min. The supernatant was lyophilized and re-dissolved by acetonitrile (containing 10% acetic acid). Each sample was filtered by a 0.22 μm membrane filter, and then detected by HPLC. The dissolved Lip of each sample was calculated according to the standard curve of Lip (ranging from 1.0 μM to 0.80 mM). The HPLC with UV detection at 270 nm for Lip. A symmetry® C18 column (5 μm, 4.6 × 250 mm) was employed. The mobile phase comprised 0.10% v/v formic acid in water (A) and acetonitrile (B), and the following gradient elution sequence was applied: 95% – 5% A (0 – 10 min), 5% – 95% A (10 – 14.10 min), and 95% A (14.10 – 20 min) at a flow rate of 1 mL/min. The chromatographic experiments were carried out at 40 °C, and the injection volume was 10 μL in all experiments.[1]

5.3 Reduction kinetics of GluAC4A.

The reduction kinetics of GluAC4A was detected by UV−Vis spectroscopy. Excess SDT (10 mM) was injected into 2.5 mL of GluAC4A solution (10 μM) and the time-dependent absorbance of GluAC4A at 420 nm at 37 °C was monitored.[2]

5.4 Release kinetics of Cy7@GluAC4A.

The release kinetics of GluAC4A was detected by fluorescence spectrometry. Excess SDT (10 mM) was injected into 2.5 mL of Cy7@GluAC4A (10/12 μM) solution and the time-dependent intensity of Cy7 at 670 nm at 37 °C was monitored.

5.5 Phase-solubility curve.

The methodology was based on the solubility variation of the guest molecule (Lip) with increasing the host molecule (GluAC4A) concentration. Briefly, same excess qualities of Lip were mixed with different ratios of GluAC4A solid in a mortar, respectively. The mixtures were grounded for 30 min and collected with 5 mL PBS in bottles. And the insoluble solid was removed by centrifugation at 13400 × g for 5 min. The supernatant was diluted with 4-fold acetonitrile solution (containing 10% acetic acid) and each sample was filtered by a 0.22 μm membrane filter, the concentrations of Lip solubilized by GluAC4A were detected by HPLC according to the standard curve ranging from 10 μM to 3.1 mM. The binding affinity (K_a) was calculated from the slope of the linear portion of the phase-solubility diagram, according to equation:[3] K_a = slope/(S_0 (1 − slope)), where S_0 is the in intrinsic solubility of Lip. The complexation efficiency (CE), which refers to the complex/free host ratio, can be obtained from the slope of the phase-solubility diagram, according to equation: CE = [GH]/[H] = slope/(1 − slope), where [GH] is the concentration of dissolved complex, [H] is the concentration of dissolved free host. CE value can be used to calculate the [G]: [H] ratio, according to equation: [G]/[H] =1/(1 + (1/CE)), where [G] is the concentration of dissolved guest, [H] is the concentration of dissolved host.

5.6 TEM measurements.

The GluAC4A or Lip@GluAC4A sample was deposited on copper grid covered with an ultrathin carbon support film and dried for 2 min at room temperature. The excess fluid was removed with a piece of filter, and the sample was negatively stained with 2% uranyl acetate for 30 s. Then the sample was air-dried for over 8 h.

5.7 Fluorescence titrations.

Fluorescence titrations were performed in PBS (10 mM, pH = 7.4). The complexations of GluAC4A with reporter dyes (Cy5-DM and Cy7) were measured by direct fluorescence titrations. A mixed solution containing known amounts of GluAC4A and dye was sequentially injected into 2.5 mL dye solution in a quartz cuvette. The dye concentration in mixed solution and cuvette are the same to keep dye concentration constant in the course of titrations. The fluorescence intensity was measured before the first addition and after every addition until a plateau was reached. By fitting the fluorescence intensity according to a 1:1 host-guest binding stoichiometry, the association constant was obtained. The fitting of data from direct titrations

was performed in a nonlinear manner, and the fitting modules were downloaded from the website of Prof. Nau's group (http://www.jacobs-university.de/ses/wnau) under the column of "Fitting Functions".

5.8 Fluorescence responses of Cy7@GluAC4A and Cy5-DM@GluAC4A upon the addition of various biologically coexisting species.

Various biological coexisting species of blood were added separately to Cy7@GluAC4A (10/10 μM) or Cy5-DM@GluAC4A (10/10 μM) in PBS (10 mM, pH = 7.4) at 25 °C and stirred for 30 min and then the fluorescence intensity of Cy7/Cy5-DM was determined. The fluorescence of Cy7/Cy5-DM alone were used as control. The biological coexisting species and their concentations used in these experiments were based on the real condition of human blood: NAD 24 μM, BSA 10 μg/mL, glutathione 8.0 μM, urea 4.0 mM, ATP 0.40 μM, glucose 5.0 mM, creatinine 80 μM, glutamine 0.50 mM, alanine 0.40 mM, glycine 0.30 mM, arginine 0.14 mM, valine 0.20 mM, lysine 0.20 mM, proline 0.20 mM, K^+ 4.5 mM, Ca^{2+} 2.5 mM, Na^+ 144 mM and Mg^{2+} 0.80 mM.[4]

5.9 Mouse model of ischemic stroke.

Young adult C57BL/6J male mice (8-10 weeks old, supplied from Beijing Vital River Laboratory Animal Technology) were maintained under controlled conditions (25 ± 2 °C, 60 - 70% humidity, 12/12-hour light/dark cycle) with access to food and water. The wire embolization transient (60 min) middle cerebral artery occlusion model was performed as previously described.[5] Briefly, mice were anesthetized with 4% isoflurane in 70% N_2O and 30% O_2, placed under a dissecting microscope. First, the skull of each mouse was exposed preoperatively and connected to a laser Doppler flowmeter (PF5001, Perimed, Switzerland), and then subjected to median incisions on the neck. The left common carotid artery was isolated, and the common and external carotid arteries were separated. A 6-0 thread was inserted into the internal carotid artery via external carotid arteries to block the middle cerebral artery. Cerebral ischemia was induced, and successful MCAO was confirmed by a reduction in cerebral blood flow exceeding 70%. 60 min after ischemia, sutures were removed, incisions closed, and mice kept warm until they regained consciousness. Sham-operated mice underwent a similar procedure without internal carotid artery insertion.

5.10 Laser speckle imaging.

Laser speckle imaging was used to assess cortical cerebral blood flow (CBF) in mice after cerebral ischemia. The cortical CBF was measured at two different time points: before MCAO and 60 min after insertion of the suture. Methods are as follows：1) The mice were anesthetized with isoflurane, placed on a 37 °C thermostatic pad; 2) The CCD camera for laser speckle

imaging was placed above the mouse head. Laser light is scattered through the brain, and the speckle contrast is used to measure CBF. The speckle contrast is then converted into a correlation time value, which is inversely proportional to the mean blood flow velocity. 3) During the laser scanning, the contrast and color of the images were adjusted, and five consecutive 2D images of the ischemic and non-ischemic hemispheres were scanned each time.

5.11 TTC staining.

To observe the area of cerebral infarction, mice were anesthetized with isoflurane, then the mice was perfused with PBS. The brain tissue was flash-frozen in the refrigerator at −20 °C for 15 min. The frozen brain tissue was cut into 5 pieces along the coronal section, each piece was about 1mm thick, and then were incubated with the concentration of 2% 2,3,5-triphenyl tetrazolium chloride (TTC) solution in a 37 °C incubator in the dark for 15–20 min. Bright red was normal brain tissue, and white was infarcted brain area. Brain slices were subsequently fixed in 4% paraformaldehyde solution and photographed 24 h later.

5.12 Assessing GluAC4A biodistribution through *in vivo* imaging.

To investigate the capability of GluAC4A to penetrate the BBB into the brain parenchyma and achieve responsive release in ischemic brain tissues *in vivo*, we utilized Cy7 labeling (λ_{ex} = 749 nm, λ_{em} = 776 nm) to visualize GluAC4A in mice subjected to MCAO. The mice were randomly divided into two groups and received intravenous injections of either 100 µL of Cy7@GluAC4A (200/200 µM, n = 9/group) or Cy7 alone (200 µM, n = 9/group) 12 h after the induction of MCAO.

At specific time intervals post-injection (3 h, 6 h, 12 h, and 24 h), the mice were anesthetized with Isoflurane (R510-22-10, RWD), and *in vivo* fluorescence imaging of Cy7@GluAC4A or Cy7 was conducted using the IVIS Lumina imaging system (Xenogen Corporation, Hopkinton, MA, USA).

For *ex vivo* distribution analysis, mice were euthanized at 3 h, 12 h, and 24 h after injection (n = 3/group/time point), and major organs (brain, heart, liver, spleen, lung, and kidney) were collected for *ex vivo* imaging. The quantification of Cy7 signals was performed using Living Image 4.3 software.

5.13 Laser confocal imaging.

Given the challenge of exciting Cy7 fluorescence in confocal microscopy, we opted for Cy5-DM as the imaging probe, which was preloaded into GluAC4A to create Cy5-DM@GluAC4A. At 12 h post-MCAO induction, we administered injections of Cy5-DM@GluAC4A (200/200 µM) or Cy5-DM alone (200 µM) to the study groups (n = 3/group). Cy5-DM (λ_{ex} = 646 nm, λ_{em}

= 662 nm) served as the label for GluAC4A to assess the capability of GluAC4A to penetrate the BBB into the astrocytes.

Brain tissue was collected 24 h after MCAO, and 5 μm cryosections were prepared for immunostaining. These sections were co-incubated with an Alexa Fluor 488-conjugated GFAP antibody (CST, USA, 1:50 dilution) to examine the distribution of Cy5-DM in astrocytes. DAPI was used to stain the cell nuclei, and fluorescence images were captured using a CLSM, followed by analysis using ImageJ software.

5.14 Experimental treatment groups *in vivo*.

All experiments were conducted in accordance with the Institutional Animal Care and Use Committee guidelines, with random group assignments. The experimental groups consisted of:

Group 1: Sham Control.

Group 2: MCAO + PBS: MCAO mice received PBS via tail vein injection one hour after MCAO.

Groups 3 through 6 received rtPA (10 mg/kg) treatment via intravenous injection one hour after the onset of MCAO.

Group 3: MCAO + rtPA: MCAO mice received rtPA treatment alone.

Group 4: MCAO + rtPA + Lip: MCAO mice received rtPA treatment with intraperitoneal administration of Lip (S7699, Selleck, TX, USA) at a dose of 10 mg/kg one hour before inducing MCAO in mice, following a previously established protocol.[6]

Group 5: MCAO + rtPA + GluAC4A: MCAO mice received rtPA treatment with tail vein injection of GluAC4A (3 mM) one hour before inducing MCAO in mice.

Group 6: MCAO + rtPA + Lip@GluAC4A: MCAO mice received rtPA treatment with tail vein injection of GluAC4A (3 mM) and Lip@GluAC4A (3 mM) one hour before inducing MCAO in mice.

Neurological function was assessed 24 h after MCAO and then euthanized, and brain tissue samples were collected for various analyses: 1) measurements of SLC7A11 and GPX4 protein levels (n = 3/group) only for group 1 - 4, 2) Immunofluorescence staining (n = 3/group) only for group 1 - 3, 3) blood brain barrier (BBB) disruption (n = 3/group). 4) Assessment of cerebral hemorrhage volume (n = 3/group). 5) Hematoxylin and eosin (H&E) staining (n = 3/group). 6) Measurements of MDA, GSH levels (n = 3/group) and GPX4 activity (n = 6/group).

5.15 Neurological behavioral score assessment.

Neurological deficits were assessed using the modified Neurological Severity Score (mNSS), as previously reported.[5a] This scoring system, ranging from 0 to 18 points, evaluated four

aspects of neurological function: motor function, sensory function, balance, and reflexes or abnormal movements. Higher scores indicated more severe injury.

5.16 Evaluating systemic toxicity of GluAC4A and Lip@GluAC4A after prolonged *in vivo* accumulation.

To thoroughly assess the potential toxic effects of GluAC4A and Lip@GluAC4A on various organs after extended *in vivo* accumulation, all animals from different groups were sacrificed on day 7 post-MCAO. Major organs, including the brain, heart, liver, spleen, lung, and kidneys, were harvested and fixed with 4% paraformaldehyde before being embedded in paraffin. Subsequently, H&E staining was performed, and the tissue sections were imaged using an optical microscope (Olympus BX51, Japan).

5.17 Lactate dehydrogenase (LDH) release detection.

According to the protocol of a LDH Detection Kit (Beyotime, Beijing, China), brain tissue was collected from each group immediately after 24 h of Lip@GluAC4A treatment. Then the brain tissue is homogenized and the supernatant is removed for testing. After adding the detection solution and incubating the sample at room temperature for 30 min, the absorbance at 490 nm was measured with a multimode microplate reader to analyze the release of LDH release.

5.18 Hemolysis assay.

For hemolysis experiments, fresh rabbit blood was collected and mixed with an anticoagulant. The blood was then centrifuged at 3000 rpm for 15 min to collect red blood cells. The hemolytic reaction was conducted in 10 mL glass tubes at 37 °C, following the mixing of equal volumes of different groups with erythrocytes. After 1 h of incubation, the mixture was centrifuged for 5 min at 5000 rpm. The optical density of the supernatant was measured at 570 nm. The absorbance of red blood cells lysed with 0.1% (by volume) Triton X-100 (assumed to be 100% hemolytic) served as a positive control, while normal saline served as the negative control. The hemolytic rates (RHR%) were calculated using the following formula:

$(Absorbance - Absorbance_{\text{background}}) / (Absorbance_{\text{maximum}} - Absorbance_{\text{background}})$

5.19 Quantification of cerebral hemorrhage.

Ischemic hemispheric brain tissues were harvested for quantifying cerebral hemorrhage using Drabkin reagent (Sigma–Aldrich). Each brain was homogenized in 1 mL Drabkin reagent mixed with 0.50 μL of 30% Brij L23 Solution and then centrifuged at 13,000 × g for 30 min. The absorbance of the supernatant at 540 nm was measured with a multimode microplate reader, and the hemorrhage volume was quantified using a reference curve derived from fresh homologous blood.

5.20 BBB disruption measurement.

To evaluate BBB disruption, we assessed Evans blue extravasation, following previously established protocols.[5b] A 2% Evans blue solution in 0.9% sterile saline was intravenously injected at a dose of 6 mL/kg after 18 h of stroke induction. Following a 6 h circulation period, mice were transcardially perfused with PBS under anesthesia. Brain tissues were collected, photographed, and then homogenized in 1.0 mL of a 50% trichloroacetic acid solution for 10 s to measure Evans blue extravasation. After centrifugation at 15,000 × g at 4 °C for 15 min, the supernatant was collected and mixed with a 4 × volume of ethanol. The content of Evans blue dye in the ischemic tissue was quantified by measuring the absorbance at 620 nm using a multimode microplate reader and determined based on a standard curve.

5.21 Double immunostaining.

To assess the expression of SLC7A11, GPX4 and COX2 in different cells within ischemic brains, paraffin-embedded brain tissue slices were collected 24 h after MCAO. These slices were incubated with anti-SLC7A11 antibody (NOVUS, USA, 1:100 dilution), anti-GPX4 antibody (Abcam, USA, 1:100 dilution) or anti-COX2 antibody (Proteintech, China, 1:50). Neurons were identified using an anti-NeuN antibody (Abcam, USA, 1:400 dilution) and astrocytes were identified using anti-GFAP and S100β antibodies (CST, USA, 1:400 dilution). The expression of SLC7A11, GPX4 and COX2 in different cell types was visualized using laser confocal microscopy (LSM 800, Zeiss, Germany).

5.22 Cell culture and hypoxia/reoxygenation experiments.

To establish an *in vitro* H/R model, we acquired the mouse brain astrocyte cell line (C8-D1A) from the American Type Culture Collection (ATCC), and 2 h oxygen-glucose deprivation (OGD) was performed according to previous study.[5a]

Primary astrocytes were isolated from the cerebral cortex of 1- to 3-day-old neonatal C57BL/6J mice. Cortical cells were digested with 0.25% trypsin-EDTA at 37 °C for 15 min and subsequently suspended in DMEM supplemented with 10% FBS. The cells were collected through repetitive pipetting and filtered through a cell strainer. Monolayer primary astrocytes were established by culturing for 7 to 10 days. The cell purity exceeded 95% after careful shaking to remove floating microglia, making them suitable for Fe^{2+} and lipid ROS assays.

5.23 *In vitro* astrocyte culture experimental groups.

Cultured astrocytes were pretreated with Lip (25 nM) and Lip@GluAC4A (25/25 nM) for 12 h before the initiation of hypoxia (2 h OGD). The experimental groups include:

1) Control (Ctrl): Control group without any treatment.

2) H/R + PBS: Astrocytes subjected to 2 h hypoxia and 12 h reoxygenation.

3) H/R + rtPA: OGD astrocytes treated with rtPA (50 μg/mL, Boehringer Ingelheim, Germany).

4) H/R + rtPA + Lip: OGD astrocytes co-treated with rtPA (50 μg/mL) and Lip (25 nM).

Cells were collected for the following assessments: 1) Cell viability assays (*n* = 6/group). 2) Measurement of GSH levels (*n* = 4/group). 3) Evaluation of GPX4 activity (*n* = 3/group). 4) Indicators of BBB disruption, including transendothelial electrical resistance (TEER, *n* = 6/group) and FITC-dextran permeability (*n* = 3/group). 5) Western blotting analysis (*n* = 3/group). 6) RT-qPCR assays (*n* = 3/group) for gene expression analysis.

5.24 Intracellular uptake of Cy5-DM@GluAC4A and hypoxia-induced response.

To investigate the intracellular uptake and release of Cy5-DM@GluAC4A in mouse brain astrocytes under hypoxia conditions *in vitro*, mouse brain astrocytes (1×10^5 cells per well) were cultured to 70%–80% confluence and treated with Cy5-DM@GluAC4A (20/20 μM) for 12 h under normoxia conditions. After three PBS rinses, cells were subjected to 2 h of inclubation with either fresh DMEM (normoxia) or glucose-free DMEM (hypoxia). Following reoxygenation, 50 μg/mL rtPA was added. After 12 h of reoxygenation, Cy5-DM entry into astrocytes was observed using laser confocal microscopy (LSM 800, Zeiss, Germany).

5.25 *In vitro* cytotoxicity assessment of GluAC4A and Lip@GluAC4A.

To assess the cytotoxicity of GluAC4A and Lip@GluAC4A, we conducted Cell Counting Kit-8 (CCK-8) assays. In brief, mouse brain astrocytes were seeded into a 96-well plate at a density of approximately 1×10^4 cells per well. Upon reaching approximately 70% confluence, the cells were exposed to different concentrations of GluAC4A (10, 25, 50, 100, 200 nM) under both normoxic and hypoxic condition. Additionally, under hypoxic conditions, cells were treated with Lip@GluAC4A at concentrations of (10, 25, 50, and 100 nM), and the subsequent changes in cell viability were assessed.

5.26 Assessment of lipid peroxidation using BODIPY and flow cytometry.

To quantify lipid reactive oxygen species (ROS), we employed the live cell analysis reagent BODIPY 581/591 C11 (D3861, Invitrogen) following the manufacturer's protocol.[6] In brief, astrocytes were incubated with BODIPY (10.0 μM) for 1 h at 37 °C. Following the incubation, the cells were harvested, washed with PBS, and subsequently resuspended in 500 μL of PBS. Cell fluorescence was acquired using flow cytometry (FACSCanto II, BD, USA) and subsequently analyzed with FlowJo software.

5.27 *In vitro* BBB model.

Astrocytes were co-cultured with endothelial cells to establish an *in vitro* BBB model, as previously reported.[10] Briefly, 10,000 astrocytes were seeded on the bottom surface of the insert for 24 h. Then, the inserts were placed in a 24-well culture plate, and complete medium (300 μL in the insert and 1 mL in the outer well) was added. After 36 h of astrocytes culture,

5000 endothelial cells were seeded on the top surface of the insert. The co-culture medium was replaced every two days for four days.

5.28 TEER and permeability assessment.

To evaluate *in vitro* BBB integrity, we employed TEER and FITC-dextran permeability measurements.[11] TEER was quantified using an EVOM2 epithelial voltammeter with the STX2 electrode set from World Precision Instruments. To obtain the final TEER result, the TEER of the blank filter was subtracted from each group's TEER value. TEER was expressed as $\Omega \times cm^2$ and calculated as TEER ($\Omega \times cm^2$) = TEER (Ω) ×surface area (0.33 cm^2).

FITC-labeled dextran (MW 40,000 Da; Sigma) was utilized for permeability assessment, following established protocols.[12] In brief, BBB permeability was determined by adding 100 μg/mL FITC-dextran in DMEM to the inner chamber of an *in vitro* BBB model. Fluorescence measurements were taken using a fluorescence enzyme marker (λ_{ex} = 485 nm; λ_{em} = 525 nm). The permeability to dextran after various treatments was expressed as a percentage relative to the control. All data were derived from a minimum of three independent experiments.

5.29 Imaging with FerroOrange.

Astrocyte cells were seeded in 35 mm confocal Petri dish (Thermo Fisher Scientific) at a density of 8,000 cells/dish. Upon reaching 70–80% confluency, cells were subjected to 6 h of hypoxia followed by 12 h of reoxygenation and treated with rtPA (50 μg/mL, Boehringer Ingelheim, Germany). Cells were then stained with FerroOrange (1 μM) in serum-free medium for 30 min at 37 °C in an incubator and washed with PBS. Digital images were captured using a laser confocal microscope (LSM 800, Zeiss, Germany) with a 60 × objective lens.

5.30 Imaging of Lipid ROS.

To assess lipid peroxidation levels, lipid ROS was measured using BODIPY 581/591 C11 (D3861, Invitrogen), a live cell analysis reagent. Astrocyte cells, seeded in 35 mm confocal Petri dishes (Thermo Fisher Scientific) at a density of 8,000 cells per dish and grown to 70–80% confluency, were treated with hypoxia for 6 h, followed by 12 h of reoxygenation, and rtPA (50 μg/mL, Boehringer Ingelheim, Germany). The cells were then incubated with the reagent at a working concentration of 2.5 μM for 30 min. Images were obtained using a laser confocal microscope (LSM 800, Zeiss, Germany) with a 60 × objective lens.

5.31 GSH and MDA assay.

Brain tissue (40 mg) was weighed and combined with 400 μL of PBS. Subsequently, the tissue was homogenized using a tissue homogenizer to create a 10% tissue homogenate. After thorough mixing, 100 μL of the tissue homogenate was collected for MDA detection, and 200 μL was collected for GSH detection. The remaining tissue homogenate underwent

centrifugation at 12,000 × g at 4 °C for 10 min. The protein content of the sample was determined using the BCA method.

Lysis was performed with 0.1 ml of PBS per million cells. After lysis, centrifuge at 10,000 - 12,000 g for 10 min to obtain a supernatant for subsequent assays. The sample lysis step should be performed in an ice bath or at 4ºC. After cell sample preparation, protein concentration can be determined using BCA Protein Assay Kit to facilitate subsequent calculation of intracellular GSH and MDA content for a given amount of protein. For GSH analysis, a total GSH/oxidized GSH assay kit (Beyotime, S0053) was employed.[7] Cortical malondialdehyde (MDA) content was measured using a lipid peroxidation MDA assay kit (Beyotime, S0131S), following the manufacturer's instructions[8] Protein concentration was determined using the BCA Protein Assay Kit (PC0020, Solarbio).

5.32 GPX4 activity assessment.

To assess the alteration of GPX4 activity, we detected GPX4 activity in brain tissue and cell samples from different treatment groups using a mouse GPX4 ELISA kit (ml057982, Mlbio) following the manufacturer's instructions.[9]

5.33 SLC7A11 knockdown in astrocytes.

To investigate the role of SLC7A11 in Lip@GluAC4A treatment, we performed SLC7A11 knockdown in mouse brain astrocytes, following the manufacturer's Lipofectamine 3000 instructions (Thermo Fisher, MA, USA). Astrocytes were transfected with 50 nM siRNA along with 1 μL of Lipofectamine 3000 in 500 μL of DMEM for 6 h. Subsequently, fresh culture medium was applied for subsequent cell experiments. The siSLC7A11 oligomers and siR-NC (as a scramble control) were sourced from Genechem Company, and their sequences are provided in Supplementary Table S1. Cultured astrocytes were transfected with either SLC7A11 siRNA (siSLC7A11) or scramble control (siR-NC) 36 h before hypoxia. Prior to hypoxia, they were pretreated with Lip@GluAC4A (25/25 nM) for 12 h. Following this, they were exposed to hypoxia for 2 h. Upon reoxygenation, the cells were treated with 50 μg/mL rtPA and Lip@GluAC4A (25/25 nM) for 12 h under normoxic conditions. The experimental groups were included:

1) siR-NC + rtPA + Lip@GluAC4A: scramble control astrocyte treated with rtPA and Lip@GluAC4A.

2) siSLC7A11 + rtPA + Lip@GluAC4A: SLC7A11 knockdown astrocyte treated with rtPA and Lip@GluAC4A.

Cell collections were performed for subsequent assessments, including measurements of SLC7A11 mRNA (*n* = 3/group) and protein levels (*n* = 3/group), GPX4 activity (*n* = 5/group), GSH levels (*n* = 4/group), TEER (*n* = 7/group), and FITC-dextran permeability (*n* = 3/group).

5.34 Western blotting.

Total protein was extracted from brain tissue and astrocytes of different experimental groups using RIPA lysis buffer. Protein concentration was determined through the BCA assay. The following primary antibodies were applied:

GPX4 (ab125066, Abcam, diluted 1:5000); SLC7A11 (26864-1-AP, Proteintech, diluted 1:1000); COX2 (12375-1-AP, Proteintech, diluted 1:1000); Occludin (#91131, CST, diluted 1:1000); β-actin (TA-09, ZSGB-BIO, Beijing, China, diluted 1:1000).

For Western blotting, anti-mouse and anti-rabbit horseradish peroxidase-conjugated secondary antibodies (Proteintech, Rosemont, USA, diluted 1:10,000) were used. Quantitative analyses of the obtained images were performed using ImageJ software.

5.35 RT-qPCR.

Total RNA was extracted from cultured astrocytes or brain tissue from different groups utilizing TRIzol (Invitrogen, 15596-026) according to the manufacturer's guidelines. Total mRNA was reverse transcribed into cDNA using a reverse transcription kit from Promega (USA). The quantification of PTGS2, GPX4, and SLC7A11 mRNA expression was performed on a Roche 480 fluorescent PCR system with an SYBR Green qPCR kit (Roche, Basel, Switzerland). The internal control was normalized to β-actin using the $2^{-\Delta\Delta CT}$ method. The RT-qPCR primer sequences can be found in Table S1.

Table S1. The RT-qPCR primer sequences.

Primers	forward primer	reverse primer
mouse SLC7A11	CTATTTTACCATCAGTGGG	ATCGGGACTGCTAATGAGAATT
mouse GPX4	ATAAGAACGGCTGCGTGGTGAAG	TAGAGATAGCACGGCAGGTCCTTC
mouse PTGS2	ATTCCAAACCAGCAGACTCATA	CTTGAGTTTGAAGTGGTAACCG
mouse β-actin	GTGACGTTGACATCCGTAAAGA	GCCGGACTCATCGTACT

5.36 Statistical analysis.

All values are presented as the mean ± standard deviation (SD). Data with a normal distribution were compared using one-way ANOVA followed by the Student-Newman–Keuls test. Means between two groups were compared using a two-tailed Student’s t-test. Significances between multiple groups were compared by One-way ANOVA. To ensure result reproducibility, a minimum of three independent experiments were conducted. Statistical analyses were carried

out using GraphPad Prism 5.0 software (GraphPad Prism Software, CA, USA). Statistical significance was set at $p < 0.05$.

6. Supporting results.

6.1 The expression of SLC7A11 and GPX4 on neurons.

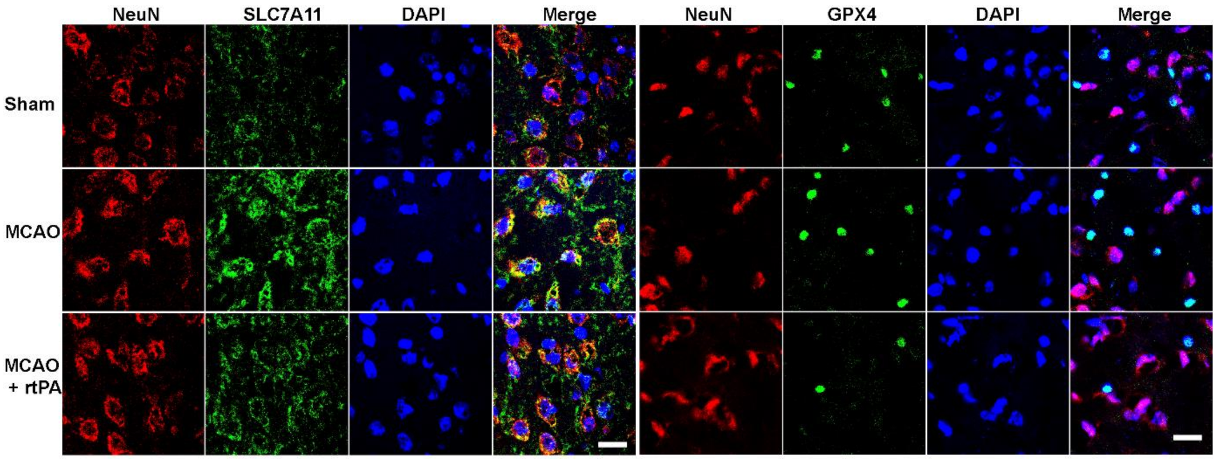

Figure S1. Immunofluorescence was used to detect the expression of SLC7A11 and GPX4 on neurons in sham-treated mice and mice at 24 h after MCAO and treatment with or without 10 mg/kg rtPA; Scale bars: 20 µm.

6.2 The expression of PTGS2 mRNA *in vivo* and *in vitro* models.

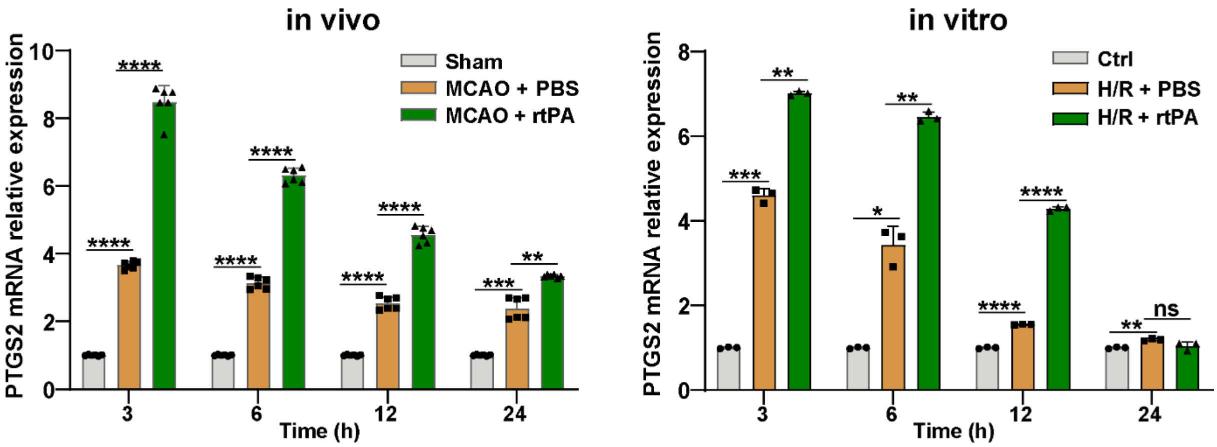

Figure S2. RT-qPCR was used to measure the mRNA level of PTGS2 in different groups *in vivo* (n = 6/group) and *in vitro* (n = 3/group) models.

6.3 The expression of COX2 in astrocytes.

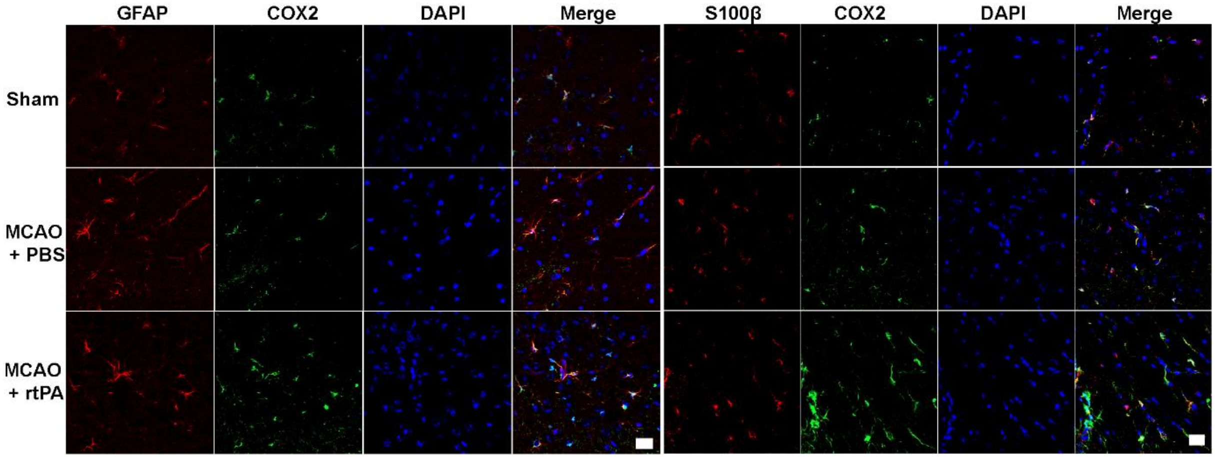

Figure S3. Immunofluorescence was employed to assess the expression of COX2 in astrocytes from sham-treated mice and mice at 24 h after MCAO with or without 10 mg/kg rtPA treatment; Scale bars: 20 μm.

6.4 Detection the critical aggregation concentration (CAC) of GluAC4A.

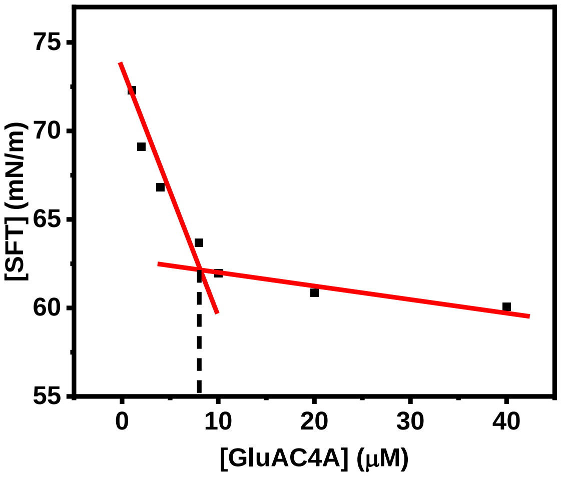

Figure S4. The CAC of GluAC4A as determined by the ring method of the surface tension meter.[13a]

6.5 Characterization of the Lip@GluAC4A co-assembly.

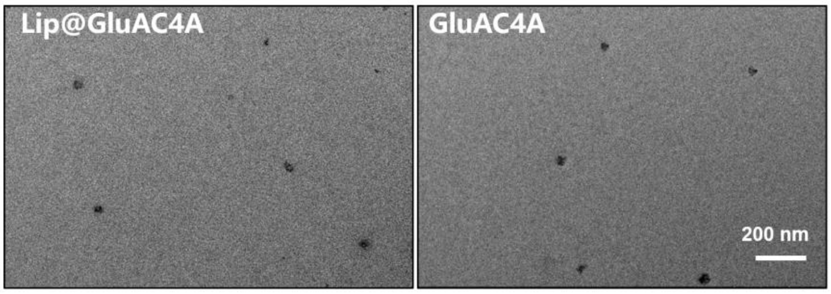

Figure S5. TEM images of Lip@GluAC4A (1 mM) and GluAC4A (1 mM). Scale bar = 200 nm.

6.6 GluAC4A improves the solubility of Lip in PBS.

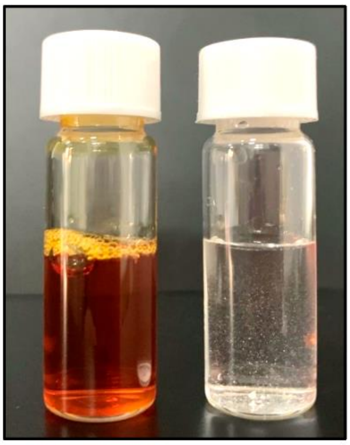

Figure S6. The picture of Lip@GluAC4A (1/1 mM, left) solution and Lip (1 mM, right) solution in PBS buffer (10 mM, pH = 7.4).

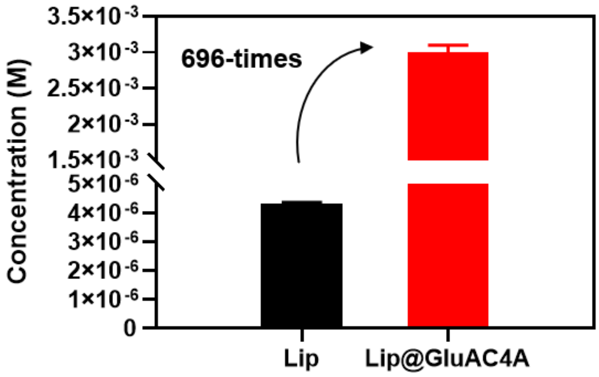

Figure S7. GluAC4A-solubilized Lip in PBS buffer (10 mM, pH = 7.4).

6.7 Phase-solubility curve.

Table S2. Parameters for phase-solubility diagrams of GluAC4A with Lip.

Host	Slope	CE[a]	[G]/[H][b]	K_a/M^{-1}[c]
GluAC4A [d]	0.998	99	99 : 100	$(3.50 \pm 0.49) \times 10^6$

[a] Complexation efficiency (CE) = [GH]/[H] = slope/(1–slope); [b] [G]/[H] = 1/(1+1/CE); [c] K_a = slope/(S_0(1–slope)); [d] The experiment was carried out in PBS solution (10 mM, pH = 7.4).

6.8 Stability testing of Lip@GluAC4A.

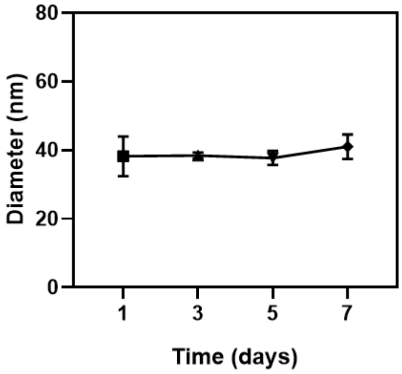

Figure S8. The stability of Lip@GluAC4A (1.0/1.0 mM) in PBS (10 mM, pH = 7.4) at 4 °C by monitoring the size change for 7 days (*n* = 3).

6.9 Reduction kinetics of GluAC4A.

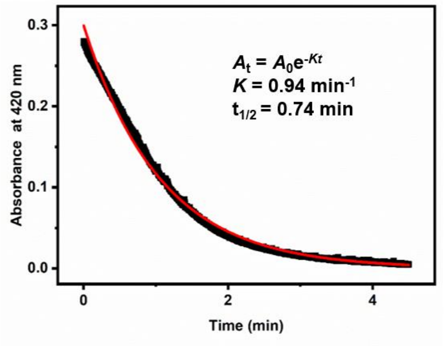

Figure S9. The reduction kinetics fitting of GluAC4A. Absorbance (black) at 420 nm of GluAC4A (10 μM) as a function of time following the addition of SDT (10 mM) and the corresponding curve (red) was fitted according to a quasi-first order reaction decay model.

6.10 Release kinetics of Cy7@GluAC4A.

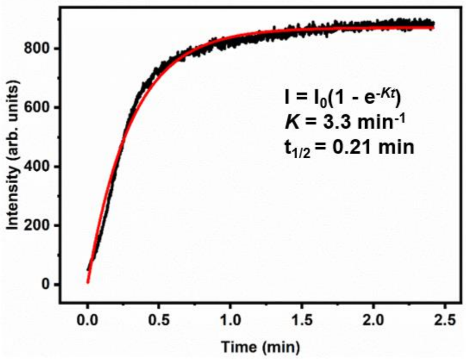

Figure S10. The release kinetics of Cy7@GluAC4A. Fluorescence intensity (black) at 760 nm of Cy7@GluAC4A (10/12 μM) as a function of time following addition of SDT (10 mM), the corresponding curve (red) was fitted according to a quasi-first order reaction model.

6.11 Binding affinities of Cy5-DM with GluAC4A.

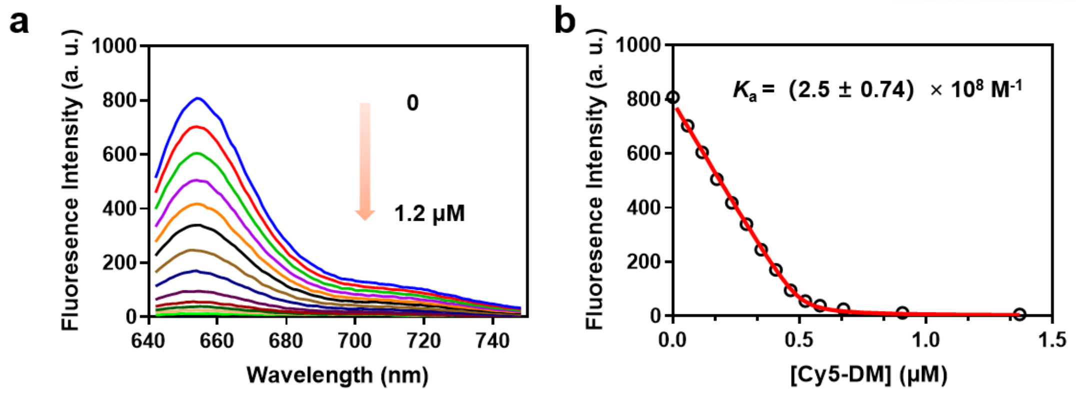

Figure S11. (a) Direct fluorescence titration of Cy5-DM (0.50 μM) with GluAC4A (up to 1.2 μM) in PBS (10 mM, pH = 7.4) at 25 °C, λ_{ex} = 625 nm. (b) The associated titration curve at λ_{em} = 654 nm was fitted according to a 1:1 binding stoichiometry.

7. References

[1] Y.-X. Yue, Z.-Z. Zhang, Z.-H. Wang, R. Ma, M.-M. Chen, F. Ding, H.-B. Li, J.-J. Li, L.-Q. Shi, Y. Liu, D.-S. Guo, *Small. Struct.* **2022**, *3*, 2200067.

[2] Z. Zhang, Y.-X. Yue, L. Xu, Y. Wang, W.-C. Geng, J.-J. Li, X.-L. Kong, X. Zhao, Y. Zheng, Y. Zhao, L. Shi, D.-S. Guo, Y. Liu, *Adv. Mater.* **2021**, *33*, e2007719.

[3] a) D. Ma, G. Hettiarachchi, D. Nguyen, B. Zhang, J. B. Wittenberg, P. Y. Zavalij, V. Briken, L. Isaacs, *Nat. Chem.* **2012**, *4*, 503; b) T. Loftsson, M. E. Brewster, *J Pharm. Sci.* **1996**, *85*, 1017.

[4] S.-H. Li, R. Ma, X.-Y. Hu, H.-B. Li, W.-C. Geng, X. Kong, C. Zhang, D.-S. Guo, *Adv. Mater.* **2022**, *34*, e2203765.

[5] a) Y. Cai, E.-Y. Yang, X.-H. Yao, X.-B. Zhang, Q.-X. Wang, Y.-F. Wang, J. Liu, W.-J. Fan, K.-K. Yi, C.-S. Kang, J.-L. Wu, *Redox. Biol.* **2021**, *38*, 101792; b) L.-N. Qiu, Y. Cai, Y.-Q. Geng, X.-H. Yao, L.-X. Wang, H.-M. Cao, X.-B. Zhang, Q.-L. Wu, D.-L. Kong, D. Ding, Y. Shi, Y.-B. Wang, J.-L. Wu, *Acta. Biomater.* **2022**, *154*, 424.

[6] Y. Cui, Y. Zhang, X.-L. Zhao, L.-M. Shao, G.-P. Liu, C.-J. Sun, R. Xu, Z.-L. Zhang, *Brain. Behav. Immun.* **2021**, *93*, 312.

[7] Y. Li, D.-C. Feng, Z.-Y. Wang, Y. Zhao, R.-M. Sun, D.-H. Tian, D.-S. Liu, F. Zhang, S.-L. Ning, J.-H. Yao, X.-F. Tian, *Cell. Death. Differ.* **2019**, *26*, 2284.

[8] X.-Y. Mu, J.-Y. Wang, H. He, Q.-F. Li, B. Yang, J.-H. Wang, H.-L. Liu, Y.-L. Gao, L.-F. Ouyang, S. Sun, Q.-J. Ren, X.-J. Shi, W.-T. Hao, Q.-M. Fei, J. Yang, L.-L. Li, R. Vest, T. Wyss-Coray, J. Luo, X.-D. Zhang, *Sci. Adv.* **2021**, *7*, eabk1210.

[9] A. Roveri, M. Maiorino, F. Ursini, *Methods. Enzymol.* **1994**, *233*, 202.

[10] F. Wang, S.-L. Ji, M.-X. Wang, L. Liu, Q.-L. Li, F.-X. Jiang, J. Cen, B.-S. Ji, *Eur. J. Pharmacol.* **2020**, *880*, 173189.

[11] Y.-H. Mi, Y.-K. Mao, H. Cheng, G.-H. Ke, M.-P. Liu, C.-P. Fang, Q. Wang, *Fitoterapia.* **2020**, *140*, 104447.

[12] R.-R. Wang, Y.-B. Zhu, Z.-W. Liu, L.-P. Chang, X.-F. Bai, L.-J. Kang, Y.-L. Cao, X. Yang, H.-L. Yu, M.-J. Shi, Y. Hu, W.-Y. Fan, B.-Q. Zhao, *Blood.* **2021**, *138*, 91.

[13] a) J.-J. Li, Y.-Q. Hu, B. Hu, W.-B. Wang, H.-Q. Xu, X.-Y. Hu, F. Ding, H.-B. Li, K.-R. Wang, X. Zhang, D.-S. Guo, *Nat. Commun.* **2022**, *13*, 6279; b) J.-J. Li, R.-X. Rong, Y. Yang, Z.-Y. Hu, B. Hu, Y.-Y. Zhao, H.-B. Li, X.-Y. Hu, K.-R. Wang, D.-S. Guo, *Mater. Horiz.* **2023**, *10*, 1689.
